# Efficient Powder Removal and Micro-Nano Structural Formation in 3D-Printed Porous Titanium Implants for Enhanced Osteogenesis

**DOI:** 10.34133/bmr.0434

**Published:** 2026-11-05

**Authors:** Jincheng Tang, Sijing Li, Yonglun Liu, Cheng Liu, Guoxian Pei, Lin Wang, Ming Yan, Dewei Zhao

**Affiliations:** ^1^ Department of Orthopedics, Affiliated Zhongshan Hospital of Dalian University, Dalian 116001, China.; ^2^ School of Materials Science and Engineering, Harbin Institute of Technology, Harbin 150001, China.; ^3^ Department of Materials Science and Engineering, Southern University of Science and Technology, Shenzhen 518055, China.; ^4^ School of Medicine, Southern University of Science and Technology, Shenzhen 518055, China.; ^5^ 3R Additive Technology (Shenzhen) Co., Ltd., Shenzhen 518055, China.; ^6^ Jiaxing Research Institute, Southern University of Science and Technology, Jiaxing 314031, China.

## Abstract

Powder bed fusion-laser beam enables the fabrication of patient-specific medical implants; however, the complete removal of residual powders from complex porous architectures remains a major challenge, compromising biological performance. In this study, a high-voltage (90 V) anodic oxidation (AO) surface polishing strategy was developed to achieve efficient powder removal and surface optimization of triply periodic minimal surface gyroid porous titanium implants. AO treatment markedly reduced surface roughness, slightly enlarged the pore size by approximately 30 to 50 μm while maintaining an overall pore size of 300 to 600 μm, and generated a honeycomb-like microstructure favorable for cell adhesion and bone ingrowth. X-ray photoelectron spectroscopy analysis confirmed the formation of a stable and compact TiO_2_ film, which enhanced hydrophilicity and negative surface charge, thereby promoting calcium phosphate deposition and osteogenic mineralization. Both in vitro and in vivo evaluations demonstrated significantly improved early-stage cell adhesion, long-term mineralization, and bone regeneration after AO treatment compared with untreated controls. Polished CP-Ti-AU-45 specimens achieved a new bone mineral density of 0.19 g/cm^3^ and a bone volume fraction of 33.8%. The molecular mechanism may involve the activation of extracellular matrix–receptor interactions, adhesion plaques, and PI3K–Akt signaling pathways while inhibiting ferroptosis-related signaling pathways, leading to a significant increase in the messenger RNA levels of Runx2 and Mef2c. The AO polishing strategy preserved the mechanical integrity of porous metals while integrating efficient powder removal with surface functionalization, thereby substantially enhancing the osseointegration potential of porous metallic implants.

## Introduction

Bone tissue engineering, proposed in the 1980s, has emerged as a highly interdisciplinary field that integrates composite materials science, cell biology, and biomedical engineering to address clinical challenges in bone repair and regeneration [1,2]. With deeper insights into the interactions between biomaterials and host tissue, the performance requirements for bone implants have evolved. Beyond the traditional emphasis on mechanical strength, hydrophilicity, degradability, and baseline biocompatibility, current demands increasingly focus on the precise control of implant geometry, biomimetic structural design, surface modification, and chemical functionalization [3,4].

Titanium and its alloys (e.g., commercially pure Ti and Ti64) are among the most widely used materials in orthopedics, dental implantation, and cardiovascular interventions due to their high specific strength, excellent corrosion resistance, and superior biocompatibility [5–7]. In physiological environments, a dense and stable TiO_2_ passive film spontaneously forms on the surface, which not only reduces metal ion release but also enhances the chemical stability and long-term biocompatibility of implants. With advances in materials science, novel titanium alloys further improve mechanical reliability and bioactivity through optimized microstructural design and surface modification, meeting increasingly complex clinical demands.

Additive manufacturing, particularly powder bed fusion-laser beam (PBF-LB), enables the precise fabrication of complex architectures by selectively melting metallic powders in a layer-by-layer manner [8]. Owing to its near-net shaping capability, high design freedom, and patient-specific customization, PBF-LB has been extensively applied in orthopedic implants such as zygomatic replacements, phalangeal prostheses, acetabular cups, and intervertebral fusion cages [9,10]. For instance, Wang et al. [11] fabricated trabecular-like acetabular cups via PBF-LB, achieving mechanical properties comparable to those of human cortical bone, thus ensuring favorable biomechanical compatibility.

The progress of precision medicine, supported by computed tomography (CT)/MRI, computer-aided design, and topology optimization, further enables the construction of highly patient-specific biomimetic porous implants through 3-dimensional (3D) printing [12]. Among these designs, triply periodic minimal surface (TPMS) architectures have gained particular attention, owing to their tunable porosity, mechanical robustness, and osteogenic potential [13]. Previous studies have demonstrated that pore sizes in the range of 300 to 600 μm are generally optimal for facilitating cell adhesion, bone ingrowth, and angiogenesis [14–16]. Considering both biomechanical and biological requirements, the gyroid configuration was selected in this study to achieve an optimal balance between biomimetic functionality and clinical feasibility.

Nevertheless, PBF-LB-fabricated porous titanium implants still face challenges such as pore-size deviation, residual stress, geometric distortion, and, most critically, residual powder adhesion. Partially melted or loosely bonded powders trapped within complex porous networks are difficult to remove completely and may detach after implantation, triggering inflammatory responses, localized bone resorption, and even mechanical failure of the implant [17]. Conventional approaches including high-pressure air blasting [18], acid etching [19], and chemical polishing [20] offer limited effectiveness in gradient porous interiors, while some may inadvertently compromise structural integrity [21]. Thus, the development of an approach capable of simultaneously achieving deep powder removal within intricate 3D pores and functionalizing the surface is of urgent importance.

Anodic oxidation (AO), a surface finishing technique based on the principle of electrochemical reaction, provides a low-stress, highly efficient, and morphology-adaptive process capable of selectively dissolving surface asperities and enabling micro/nanoscale structural regulation [22–24]. Conventional AO at relatively low voltages is primarily applied to fabricate ordered nanotubular oxide layers, which exhibit bioactive properties in vitro but often suffer from fragile walls and weak interfacial bonding, thereby limiting their long-term clinical stability.

This study unconventionally explores AO by employing elevated voltages, tailored electrolyte composition, and optimized anodization duration; efficient polishing and functional modification of both external and internal surfaces of complex porous structures were achieved. By systematically evaluating changes in the surface topography and mechanical properties of 3D-printed gyroid porous titanium before and after high-voltage AO, the strategy aims to remove residual powder without compromising mechanical integrity while establishing an osteoconductive interface. Furthermore, in vitro studies assessed osteoblast adhesion, proliferation, and differentiation, whereas in vivo experiments elucidated bone regeneration and integration mechanisms. Collectively, this work provides a novel and clinically relevant surface treatment strategy for porous titanium implants, offering strong promise for their future translational applications.

## Materials and Methods

### Design of the gyroid porous specimens

Inspired by the trabecular bone structure, a gyroid architecture was selected owing to its topological similarity and superior mechanical compatibility. Previous studies have demonstrated that gyroid structures exhibit enhanced mechanical compatibility. Early investigations indicated that pore sizes ranging from 300 to 600 μm are favorable for osteoblast proliferation and mineralization [9]. Considering the printing resolution of the SLM 125HL system, the unit cell dimensions of the porous scaffold were set to 2.5 mm × 2.5 mm × 2.5 mm with a porosity of 70%, ensuring a fully interconnected porous network. Tensile and compressive specimens were designed in accordance with the standard protocols reported by Tang et al. [25]. Cylindrical porous samples (Φ10 mm × 4 mm) were prepared for in vitro biological assays, while smaller cylindrical scaffolds (Φ3 mm × 3 mm) were fabricated for in vivo implantation in rat femoral defect models and subsequent osteogenesis evaluation.

### Fabrication of porous specimens

The porous specimens were fabricated using gas-atomized powders with particle sizes ranging from 15 to 53 μm on an SLM 125HL system equipped with a 400-W IPG fiber laser. A schematic of the printing process is shown in Fig. S1, where SDx and SDy represent laser scanning directions and BD denotes the build direction. A stripe scanning strategy was employed, with the scanning direction rotated by 66.67° between successive layers to enhance microstructural homogeneity and part quality. Among the numerous parameters affecting part quality during PBF-LB, the volumetric energy density (VED) is a critical factor, defined as

$$\mathrm{VED}=\frac{P}{v\times h\times l}$$ (1)

where *P* represents laser power, *v* is the scanning speed, ℎ is the hatch spacing, and 𝑙 is the layer thickness.

To systematically investigate the tensile and compressive properties of the porous scaffolds, process parameters were varied according to Table S1, and specimens were denoted by their corresponding VED values. All builds were carried out under a high-purity argon atmosphere to minimize oxidation. Porous gyroid scaffolds used for in vitro and in vivo experiments were printed using the optimized parameters derived from mechanical testing.

### Surface polishing of porous specimens

During PBF-LB fabrication, residual powders (both partially melted and loosely bonded particles) tend to adhere to the internal and external surfaces of porous scaffolds. To remove these residues, the gyroid specimens were first subjected to ultrasonic vibration at 25 kHz for 15 min, which effectively dislodged loosely attached powders. Subsequently, the specimens were further treated using a self-designed AO-assisted ultrasonic polishing system for the removal of residual powders. Based on the high-voltage AO principle, a relatively uniform oxide film with weak interfacial bonding is first formed on both the inner and outer surfaces of the porous material in the reactor under the combined action of the electrolyte and an externally applied electric field (Fig. S2). During the AO process, the magnetic stirring device continuously mixes the electrolyte, thereby enhancing the oxidation efficiency and suppressing local overcorrosion, while the cooling system maintains a stable reactor temperature to ensure a uniform and controllable reaction process. After the oxidation treatment, the low-frequency ultrasonic device is activated to fracture and peel off the loose oxide layer through ultrasonic vibration, thereby effectively removing adhered powder particles, particularly those trapped within porous channels.

High-voltage AO (90 V) was applied to further polish the surface and improve chemical purity. The electrolyte consisted of 94% ethylene glycol, 6% deionized water, and 0.5 wt% ammonium fluoride. During AO treatment, the scaffold acted as the anode and a graphite rod as the cathode, with a circulating water system maintaining the temperature below 40 °C. The AO treatment lasted 50 min, after which specimens were subjected to another 15 min of ultrasonic cleaning to remove loosely attached nanotubes and residual powders. Samples treated only by AO were labeled as “A”, whereas those subjected to AO followed by ultrasonic cleaning were denoted as “AU”. These suffixes were added to “CP-Ti” or “Ti64” to distinguish them from untreated controls. For biological assays, all scaffolds were sterilized in an autoclave at 120 °C for 20 min.

### Mechanical testing and surface characterization

Tensile and compressive tests were performed using an Instron 68TM-30 dual-column electronic testing system with a maximum load capacity of 30 kN, equipped with an AVE 2 extensometer for optical strain measurement. The loading rate was 0.2 mm/min. Tensile tests were terminated when stress dropped by more than 50% or when fracture occurred, while compressive tests were stopped when stress decreased by more than 50% or when specimens reached near-dense states. High-resolution digital microscopy was employed for real-time monitoring of deformation. Each condition was tested with at least 5 replicates, and statistical analysis was performed to ensure reproducibility.

Macroscopic and metallographic morphologies were examined using an MCK-50MC optical microscope, and relative density was quantified with the ImageJ software. Surface roughness before and after polishing was assessed using a Bruker Contour GT-K 3D profilometer, and surface topography was reconstructed in 3 dimensions. Microstructural analysis was carried out using a ZEISS Merlin scanning electron microscope (SEM) operated at 5 kV and 100 pA. Electron backscatter diffraction was performed with an accelerating voltage of 20 kV, a probe current of 5 nA, and a step size of 0.2 μm. Surface chemical composition and oxidation states were analyzed using x-ray photoelectron spectroscopy (XPS; Escalab Xi+) with 4-keV Ar^+^ ion sputtering at a rate of ~0.3 nm/s for depth profiling. Spectra were analyzed qualitatively and quantitatively using the Avantage software.

### Cell culture

MC3T3-E1 preosteoblasts and MG-63 (ATCC) were cultured in 25-cm^2^ tissue culture polystyrene flasks using high-glucose Dulbecco’s modified Eagle medium supplemented with 10% fetal bovine serum (Gibco) and 1% penicillin/streptomycin. Cells were maintained in a humidified incubator (HF90, Heal Force) at 37 °C with 5% CO_2_ and 100% humidity. The medium was refreshed every 3 d.

### Cytocompatibility evaluation

MC3T3-E1 cells were seeded onto porous cylindrical scaffolds in 24-well plates at a density of 5 × 10^4^ cells/well. After 2 h of incubation, the medium was replaced, and samples were washed 3 times with phosphate-buffered saline (5 min each). Cells were fixed with 0.25% glutaraldehyde at 4 °C for 12 h, followed by dehydration in a graded ethanol series (10% to 100%, 10 min each). Samples were air-dried for 24 h, sputter-coated with gold, and examined under an SEM to assess adhesion.

For proliferation assays, cells were seeded at the same density and cultured for 1, 3, and 5 d. Cell viability was evaluated using Cell Counting Kit-8 (CCK-8) (Shanghai Yisheng Biotech). At each time point, 10% (v/v) CCK-8 solution was added, and samples were incubated at 37 °C for 1 h. Absorbance was measured at 450 nm using a microplate reader (IMark, Bio-Rad).

### Osteogenic differentiation assessment

MG-63 cells co-cultured with scaffolds for 7 and 14 d were lysed with 1% Triton X-100 using freeze–thaw cycles between −80 and 4 °C. Protein content and alkaline phosphatase (ALP) activity were quantified using bicinchoninic acid and ALP assay kits (Solarbio), respectively.

For long-term osteogenesis evaluation, extracellular matrix (ECM) mineralization was assessed via alizarin red S (ARS) staining. MG-63 cells (4 × 10^4^ cells/well) were seeded onto porous scaffolds and cultured overnight before being transferred to an osteogenic medium containing 10 mM β-glycerophosphate, 0.3 mM ascorbic acid, and 100 nM dexamethasone. After 21 d, samples were fixed in 4% paraformaldehyde, stained with 2% ARS (pH 4.3) for 5 min, and rinsed with deionized water. Stained mineral deposits were visualized by optical microscopy. Bound calcium was subsequently eluted with 10% cetylpyridinium chloride for 15 min, and absorbance was measured at 562 nm.

### Transcriptomic analysis

Polished porous samples were co-cultured with MC3T3-E1 cells for 72 h. After incubation, the culture medium was removed, and the cells were washed twice with phosphate-buffered saline. Subsequently, 400 μl of RNAiso Plus reagent (Takara, Japan) was added to each well and incubated on ice for 5 min. The cells were repeatedly pipetted using RNase-free tips until the lysate became viscous and transparent and then collected into 1.5-ml RNase-free microcentrifuge tubes.

Total RNA was extracted according to the standard RNAiso Plus protocol, including chloroform extraction, isopropanol precipitation, and washing with 75% ethanol. After air-drying, the RNA pellet was dissolved in 20 μl of RNase-free water. RNA concentration (≥150 ng/μl) and purity (A260/280 ratio of 1.9 to 2.1) were measured using a NanoDrop 2000 spectrophotometer. RNA integrity was evaluated using an Agilent 2100 bioanalyzer, and samples with an RNA integrity number ≥8.0 were considered qualified. The qualified RNA samples were stored at −80 °C and subsequently subjected to transcriptome sequencing on the Illumina NovaSeq 6000 platform.

The quality of the raw sequencing data was assessed using the FastQC software (http://www.bioinformatics.babraham.ac.uk/projects/fastqc/), including evaluation of base quality score distribution, per-base sequence quality, GC content, polymerase chain reaction duplication levels, and *k*-mer frequency. Gene expression levels were calculated using the RPKM (reads per kilobase per million mapped reads) method. Differential expression analysis was performed using DESeq2, with the screening criteria set as fold change (FC) > 1.5 and false discovery rate (FDR) < 0.05. The results of differential expression analysis were visualized using the ggplot2 package. Gene Ontology (GO) functional enrichment analysis of differentially expressed genes (DEGs) was conducted using the clusterProfiler package.

### Animal model and implantation

All animal experiments were approved by the Institutional Animal Care and Use Committee of the Southern University of Science and Technology (Approval No. SUSTech-SL2024040201). Healthy 8-week-old specific-pathogen-free-grade male Sprague–Dawley rats (350 to 400 g) were randomly divided into 5 groups (*n* = 3 per group): blank control, CP-Ti-45, CP-Ti-AU-45, Ti64-45, and Ti64-AU-45. The number of rats used in each group was determined with reference to previous literature [26,27].

Femoral implantation was performed under sterile conditions. Rats were anesthetized with isoflurane (maintenance at 3%) and administered subcutaneous meloxicam (1 μl/g body weight) for analgesia. Following shaving and disinfection, a 10 × 5 mm incision was made above the distal femoral metaphysis. A cylindrical bone defect (Φ3 mm × 3 mm) was created using a drill, and bone debris was flushed with sterile saline. Sterilized implants were press-fitted into the defects, and the incision was sutured in layers. Penicillin (6,000 U/kg) and carprofen (4 mg/kg) were administered postoperatively for 3 d. Rats were euthanized after 4 weeks, and femurs with implants were harvested for micro-CT and histological evaluation.

### Micro-CT analysis

Excised femurs were fixed in 4% paraformaldehyde and scanned using a Scanco μCT100 system under the following conditions: 90 kV, 200 μA, 20-μm voxel size, and 300-ms exposure. Three-dimensional reconstruction of implants and surrounding bone within 500 μm was performed using the Scanco software, and bone morphometric parameters were quantified with CTAn, including bone–implant contact (BIC), bone mineral density (BMD), bone volume fraction (BV/TV), trabecular spacing (Tb.Sp), trabecular number (Tb.N), and trabecular thickness (Tb.Th).

### Histology and immunohistochemistry

Fixed femurs were processed for undecalcified histology (*n* = 3 per group). After dehydration in graded ethanol and clearing in xylene, samples were embedded in polymethyl methacrylate. Sections were cut longitudinally perpendicular to the implant axis, yielding 4 to 5 slices per femur, each polished to ~100-μm thickness. Methylene blue, toluidine blue, and Van Gieson (VG) staining were performed, and quantitative analyses were carried out using ImageJ. The region within 250 μm of the implant was defined as the region of interest.

### Schematic diagram of the overall study

The schematic diagram of the high-voltage AO strategy for the highly efficient removal of residual powders from the gyroid structure and the resulting improved biocompatibility is shown in Fig. 1.

**Fig. 1.**
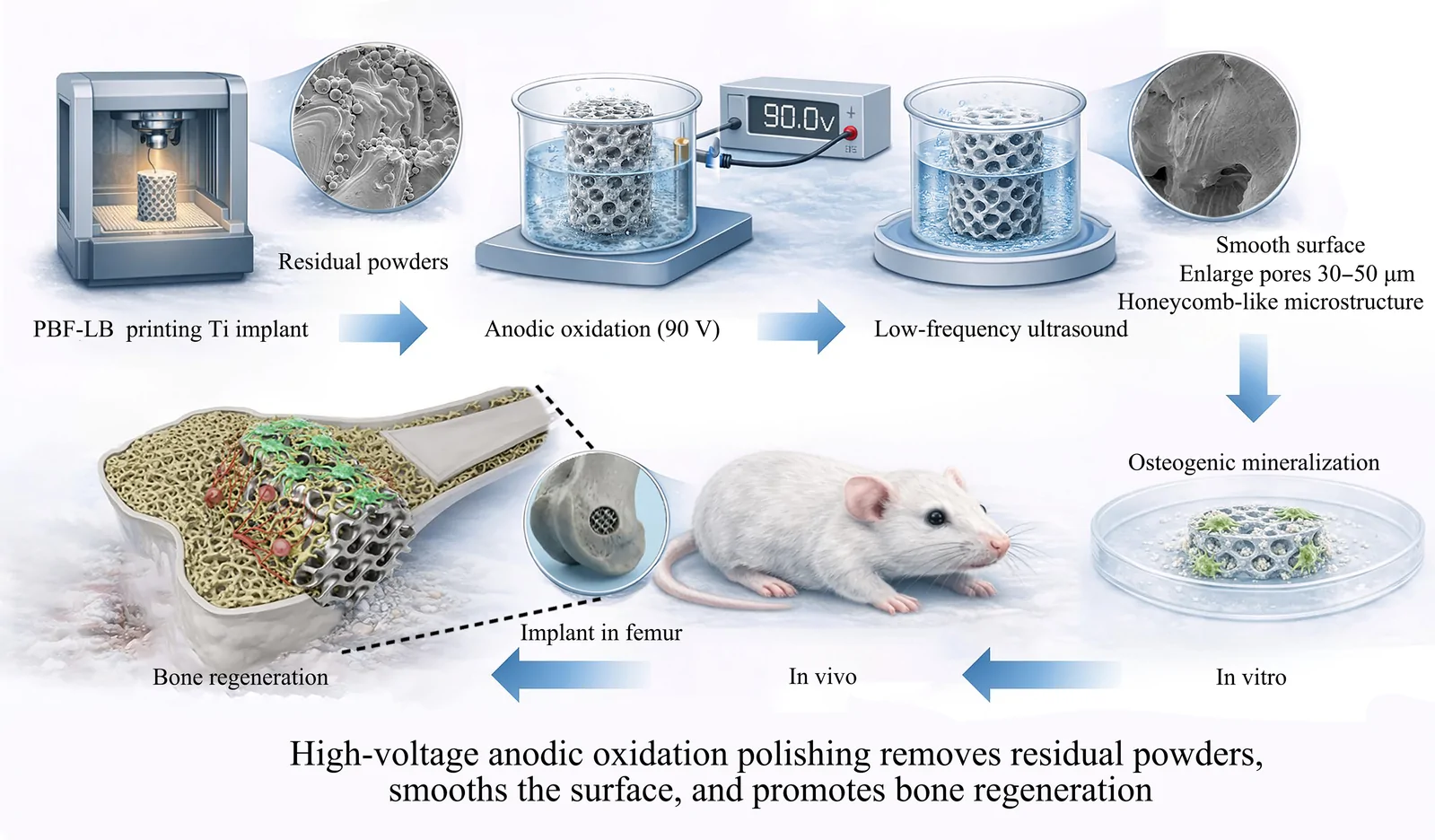
Schematic diagram of the efficient residual powder removal and enhanced bioactivity following surface polishing of gyroid porous Ti implants.

### Statistical analysis

All experimental data are presented as mean ± standard deviation from at least 3 independent replicates. Statistical significance was determined by one-way analysis of variance, with *P < 0.05 considered significant.

## Results

### As-printed porous Ti metals prior to polishing/removal treatment

During the optimization of PBF-LB processing parameters for fabricating gyroid-structured porous Ti alloys, it was observed that insufficient laser power (e.g., CP-Ti-35 and Ti64-35) led to pronounced structural defects within the specimens. These defects primarily originated from an inadequate VED, which failed to achieve full melting and bonding of powders, resulting in porosity and structural discontinuities (Fig. 2A and B). With increasing laser power, such defects gradually diminished, and the relative density of the samples improved, indicating that a higher VED facilitated more complete melt pool consolidation. At higher power levels, the relative densities of CP-Ti-50 and Ti64-50 specimens reached 99.85% and 99.77%, respectively (Table S2). At a VED of 45 J/mm^3^, CP-Ti-45 and Ti64-45 achieved relative densities of 99.82% and 99.74%, comparable to that of the 50 J/mm^3^ group, suggesting that densification had already plateaued within this range. Nevertheless, a small number of residual pores were still detectable, most likely arising from trapped gas bubbles during rapid solidification or from incomplete gas escape prior to full solidification of the melt. Furthermore, increasing VED elevated both surface and bulk temperatures, which could destabilize the melt pool, induce localized overmelting or hot cracking, and ultimately compromise printing accuracy and mechanical performance.

**Fig. 2.**
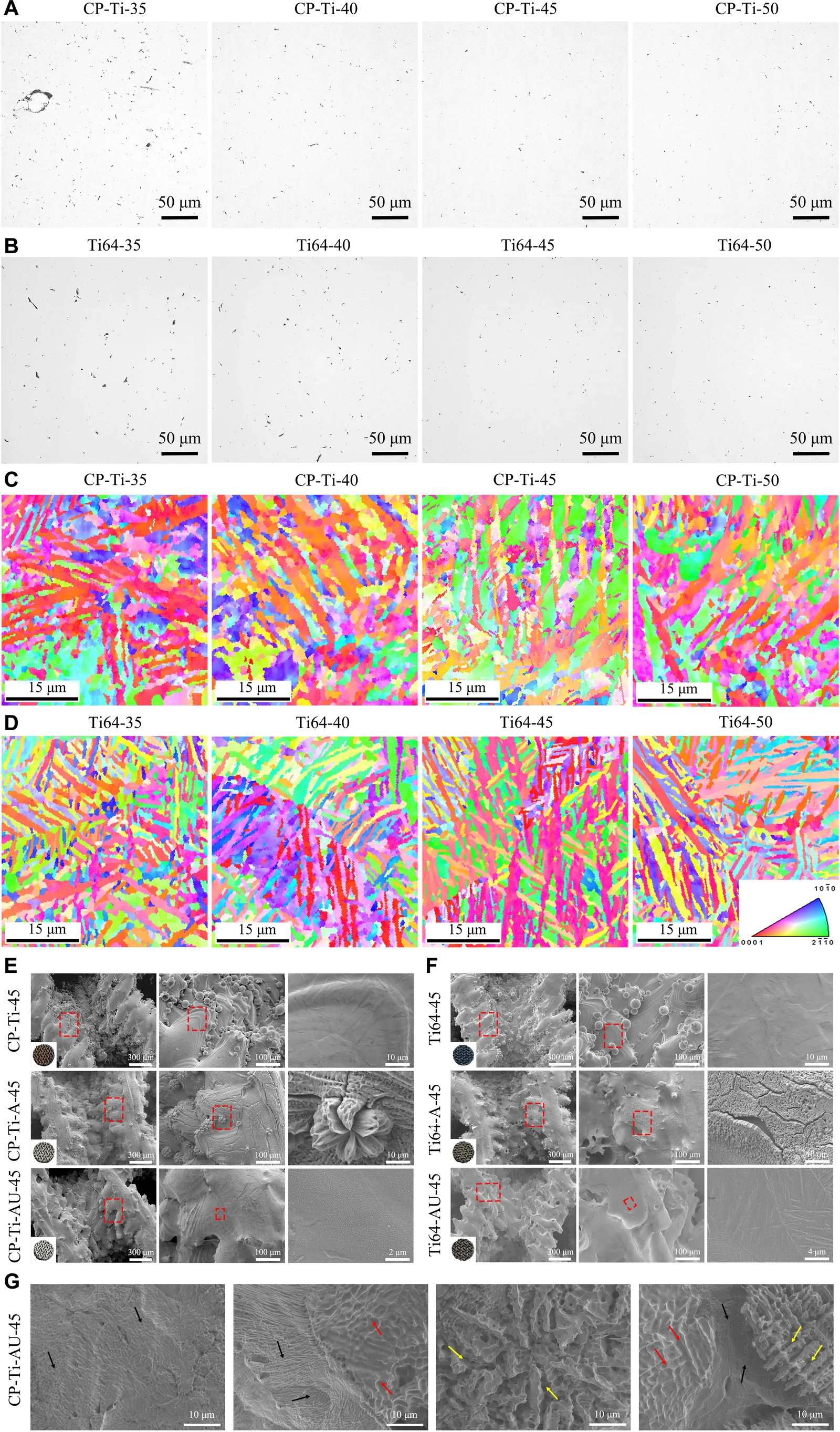
Optical micrographs of gyroid porous (A) CP-Ti and (B) Ti64 samples under different printing parameters. Electron backscatter diffraction (EBSD) inverse pole figure maps of gyroid porous (C) CP-Ti and (D) Ti64 samples under different printing parameters. SEM images of the surface morphology of gyroid porous (E) CP-Ti and (F) Ti64 samples under different printing parameters. (G) SEM images of honeycomb-like (black arrows), honeycomb serrated (red arrows), and Haversian tubular (yellow arrows) structures on the surface of the gyroid porous CP-Ti-AU-45 sample.

Grain size analysis (Fig. 2C and D and Table S2) revealed that larger VED values promoted recrystallization and grain coarsening, owing to enhanced thermal input. A higher VED not only reduced porosity and unmelted particles but also improved density and geometric accuracy. However, an excessive VED risked excessive grain growth, structural inhomogeneity, and potential degradation of anisotropic mechanical properties. Thus, the VED must be carefully optimized to balance melt pool stability and microstructural uniformity [28].

Due to the ultrahigh cooling rate characteristic of the PBF-LB process (10^3^ to 10^8^ K/s) [29], refined α′-Ti grains developed as layered microstructures with serrated morphologies. Electron backscatter diffraction results clearly demonstrated that as VED increased from 35 to 50 J/mm^3^, the average grain size of CP-Ti enlarged from 2.60 to 3.26 μm. The grain morphology evolved from initially fine, equiaxed, and randomly oriented grains to lamellar or elongated structures with distinct directional features (Fig. 2C). At a lower VED, rapid solidification and smaller melt pool dimensions restricted grain growth, yielding a relatively homogeneous and equiaxed microstructure. In contrast, a higher VED prolonged the thermal exposure and slowed the cooling rate, favoring preferential growth of nuclei along energetically favorable orientations, thereby generating directionally oriented lamellar structures.

For Ti64 specimens, the average grain size increased more modestly from ~2.25 μm at 35 J/mm^3^ to ~2.52 μm at 50 J/mm^3^, while the microstructure evolved from near-equiaxed grains toward elongated and lamellar morphologies (Fig. 2D). Enhanced thermal input promoted α′ lamellar coarsening, producing more pronounced texture and distinct phase boundaries. Compared with the fine and homogeneous microstructure under a low VED, high-VED conditions induced texture strengthening, with grains of similar orientation tending to cluster and form directionally aligned structures. Unlike CP-Ti, Ti64 intrinsically contains both α and β phases, making its phase transformation kinetics more complex and thus more prone to layered lamellar structural transitions. This phenomenon may influence the anisotropic mechanical response of the alloy.

### Surface morphology characteristics of porous Ti metals after polishing

The as-printed CP-Ti-45 exhibited relatively smooth top surfaces due to uniform melt pool solidification, whereas the sidewalls were rough owing to partially fused powders. After anodization, a 15- to 30-μm-thick TiO_2_ nanotube array formed on the surface, accompanied by local delamination and cracks. Nanotubes also developed on adhered powders at the sidewalls, producing “popcorn-like” surface features. Subsequent anodic oxidation polishing (AO polishing) effectively removed loosely bound powders, producing interconnected and well-defined pores with reduced roughness while retaining hierarchical micro/nanostructures (Fig. 2E). At higher magnifications, 3 distinct features were observed: (a) honeycomb-like patterns exposed after nanotube detachment, which provide additional anchoring sites and nutrient reservoirs; (b) firmly bonded powder remnants exhibiting “serrated honeycomb” pits that facilitate cytoskeletal anchoring; and (c) partially fused particles with “Haversian tubular” ridges, potentially serving as guiding channels for cell alignment and nutrient transport (Fig. 2G). The overall pore size increased by 30 to 50 μm after polishing, enhancing pore interconnectivity.

Ti64 exhibited a similar but less stable evolution. After anodization, the nanotube layer was discontinuous, undergoing a “growth–delamination” process that resulted in a smaller increase in surface roughness compared to CP-Ti (Fig. 2F). AO polishing removed residual powders and reduced roughness, but large-scale delamination was occasionally observed, likely due to local embrittlement and ultrasonic stress. Nevertheless, no marked deterioration in bulk mechanical properties was detected. Comparatively, CP-Ti retained more ordered and intact micro/nanostructures with superior pore connectivity, whereas Ti64 was more susceptible to structural disruption during polishing.

Three-dimensional roughness measurements further confirmed these observations (Fig. S3). The initial average surface roughness was 1.2 to 1.6 μm. After anodization, CP-Ti-A-45 reached 1.94 μm due to nanotube growth and partial delamination, markedly higher than Ti64. AO polishing removed weakly adhered nanotubes and powders, reducing roughness below the as-printed state while preserving part of the micro/nanoframework. For instance, CP-Ti-AU-45 displayed a smoother surface yet retained well-defined nanoarchitectures, which are expected to enhance hydrophilicity and initial cell adhesion.

To elucidate the chemical evolution of surface layers, XPS depth profiling was performed (Fig. 3A to C). The as-printed CP-Ti-45 showed a thin native TiO_2_ layer, with metallic Ti signals emerging after 20-s sputtering. Anodization yielded a thicker and denser TiO_2_ film (with 2p_3/2_ and 2p_1/2_ peaks at 458.5 and 464.2 eV, respectively), maintaining high oxygen levels even after 80 s. AO polishing removed weakly bound nanotubes, allowing Ti metal (with 2p_3/2_ and 2p_1/2_ peaks at 454.1 and 460.1 eV, respectively [30]) to appear after 20 s, yet the stable underlying oxide film remained intact (Fig. 3G).

**Fig. 3.**
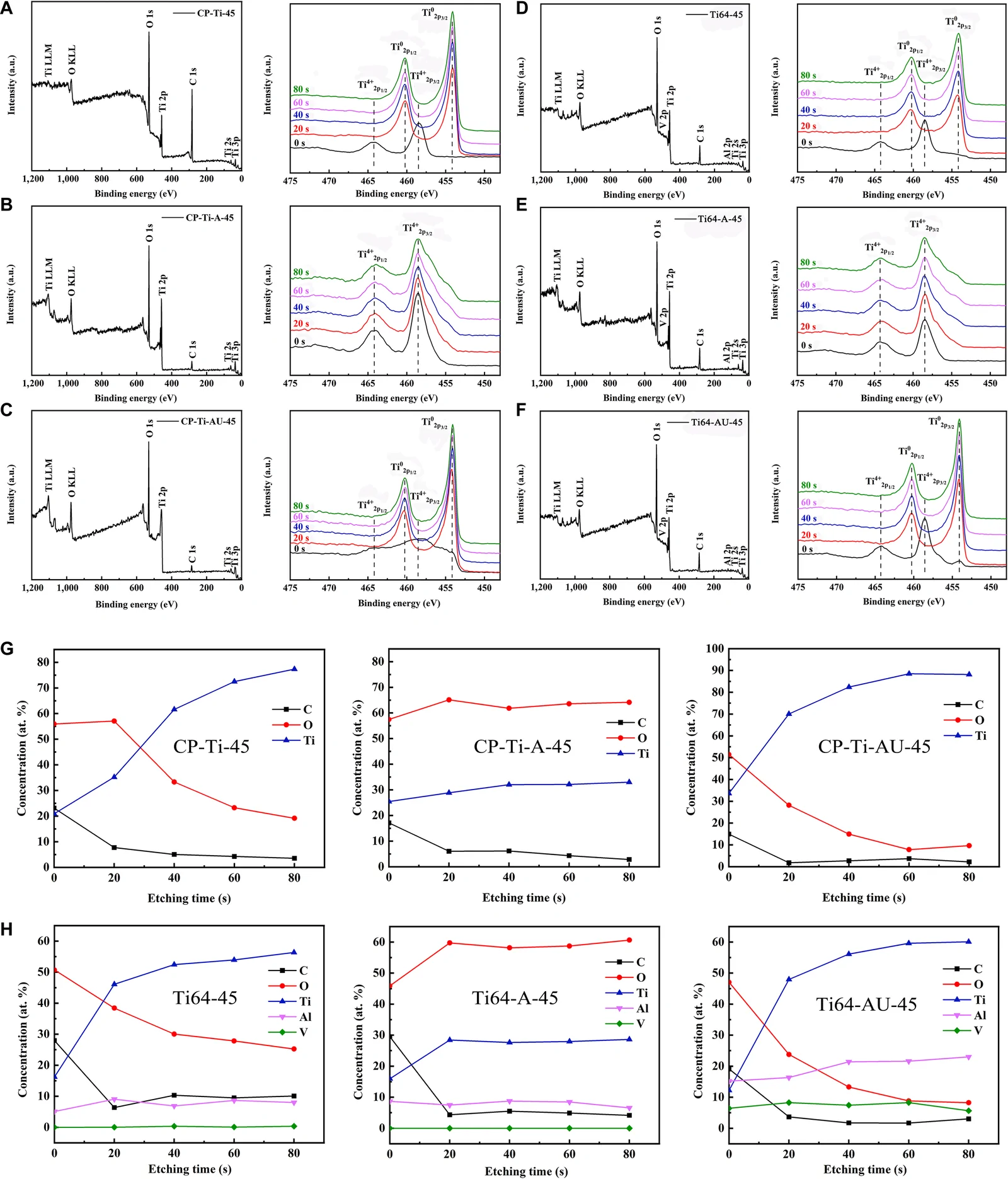
X-ray photoelectron spectroscopy (XPS) survey spectrum and detailed XPS spectra of Ti on the surface of gyroid porous (A) CP-Ti-45, (B) CP-Ti-A-45, (C) CP-Ti-AU-45, (D) Ti64-45, (E) Ti64-A-45, and (F) Ti64-AU-45 samples. Element concentration vs. etching time of gyroid porous (G) CP-Ti and (H) Ti64 samples, sputtered at approximately 0.3 nm/s.

Ti64 exhibited a comparable trend, but the presence of alloying elements (Al and V) introduced distinct features (Fig. 3D to F and Fig. S4). In Ti64-A-45, metallic Ti and Al (with a 2p peak at 72.6 eV [31]) signals emerged after ~40 s, while metallic V (with 2p_3/2_ and 2p_1/2_ peaks at 512.3 and 519.7 eV, respectively [32]) remained undetectable even after 80 s, indicating effective encapsulation by the oxide film (Fig. S4B). After AO polishing, both CP-Ti and Ti64 surfaces were oxygen-rich with rapid transitions to the metallic substrate (Fig. S4C). Semiquantitative analysis revealed codistribution of Ti, Al, and V across the oxide-to-metal interface, reflecting alloy-specific interfacial evolution (Fig. 3H).

In summary, AO polishing substantially optimized the surface of porous titanium alloys without compromising bulk mechanical integrity. CP-Ti exhibited more ordered and stable micro/nanoarchitectures, lower roughness, and a denser TiO_2_ film, whereas Ti64 showed reduced structural retention due to the influence of Al and V. These modifications collectively provide favorable physicochemical conditions for improved hydrophilicity, cytocompatibility, and long-term interfacial stability of 3D-printed Ti implants.

### Compressive mechanical properties of porous Ti metals after polishing

To evaluate the influence of high-voltage AO polishing on the compressive behavior of additively manufactured porous metals, a systematic comparison was conducted between unpolished and AO-polished (-AU) gyroid-structured CP-Ti and Ti64 specimens fabricated under different VED conditions (35 to 50 J/mm^3^). The analysis considered yield strength, ultimate compressive strength, strain at failure, plateau stress (CP-Ti only), and energy absorption per unit volume.

Plateau stress refers to the relatively constant stress response exhibited by a material within a defined strain interval during compression. Notably, Ti64 specimens displayed brittle behavior with premature failure and lacked a well-defined plateau region; therefore, plateau stress values were not systematically recorded for Ti64.

Energy absorption per unit volume is a critical metric for evaluating the damping and cushioning capacity of TPMS porous structures in orthopedic implants, particularly at high porosity where large-deformation capacity enhances energy dissipation. For the gyroid structures investigated, this parameter was calculated as follows:

$$W=\int_{0}^{\varepsilon_{d}}\sigma \left(\varepsilon \right)\mathrm{d\varepsilon}\approx \sum_{i=1}^{n}\frac{\sigma_{i}+\sigma_{i-1}}{2}\left(\varepsilon_{i}-\varepsilon_{i-1}\right)$$ (2)

where *W* is the absorbed energy per unit volume (MJ/m^3^), *ε_d_* is the target strain (50% in this study), and *σ* is the compressive stress (MPa).

The absorbed energy corresponds to the area enclosed between the stress–strain curve and the strain axis. For CP-Ti, evaluations were conducted at 50% strain, whereas Ti64 specimens failed before reaching this strain and were therefore excluded from this calculation.

With increasing VED (35 to 50 J/mm^3^), both CP-Ti and Ti64 exhibited improved densification and metallurgical bonding, reduced porosity, and enhanced structural stability (Fig. 4A and B). At 50 J/mm^3^, CP-Ti-50 achieved a yield strength of 113.7 MPa, ultimate compressive strength >2,000 MPa, plateau stress of 207.4 MPa, energy absorption of 113.1 MJ/m^3^, and strain at failure exceeding 80%. However, CP-Ti-45 also maintained an ultimate compressive strength >2,000 MPa and a strain at failure >80%, with other mechanical parameters remaining close to those of CP-Ti-50 (Fig. 4C and Table S3). Thus, increasing VED from 45 to 50 J/mm^3^ provided only limited additional mechanical benefits.

**Fig. 4.**
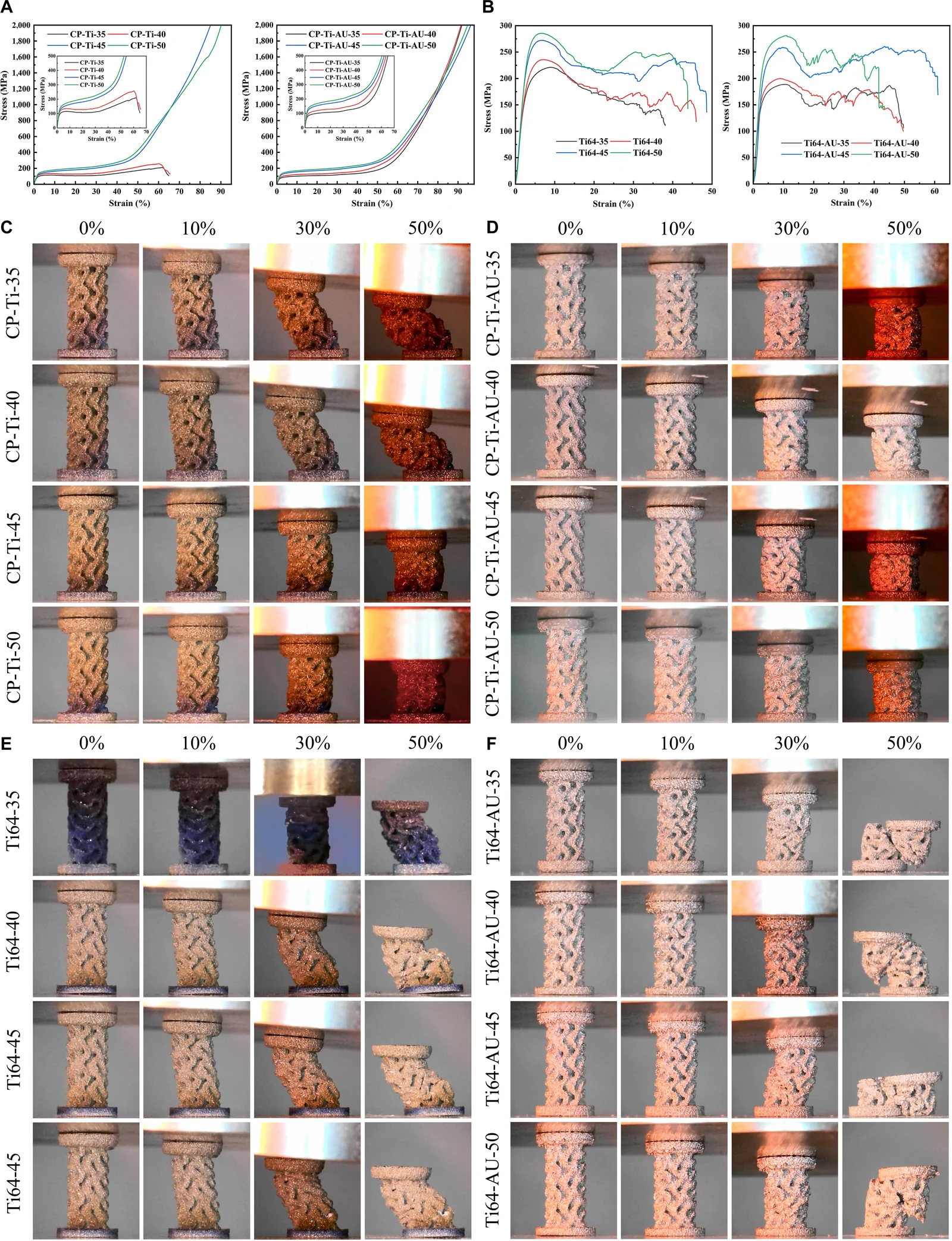
Compressive stress–strain curves of gyroid porous (A) CP-Ti and (B) Ti64 samples under different printing parameters. Morphological images of gyroid porous (C) CP-Ti, polished (D) CP-Ti-AU, (E) Ti64, and polished (F) Ti64-AU samples at different deformation stages (0%, 10%, 30% and 50%) during compressive loading.

Under the same condition, Ti64-50 reached 234.3 MPa yield strength, 285.6 MPa ultimate strength, and 102.4 MJ/m^3^ energy absorption, with a failure strain of 43.84% (Fig. 4E and Table S4). At 45 J/mm^3^, both alloys demonstrated a favorable balance between strength and deformation tolerance: CP-Ti-45 maintained >2,000 MPa ultimate strength and >80% strain at failure, while Ti64-45 exhibited enhanced energy absorption (106.6 MJ/m^3^) and strain at failure (48.67%). Although Ti64-50 showed higher strength, Ti64-45 provided greater energy absorption and deformation capacity, indicating that the highest VED did not necessarily produce the most favorable overall mechanical response.

AO polishing markedly modified surface morphology by removing residual powders and unstable oxide films, resulting in a pore-size enlargement of approximately 30 to 50 μm, reducing roughness and alleviating stress concentration. Importantly, the mechanical effect of AO polishing was governed by a balance between slight changes in load-bearing strength and improved deformation capacity. For CP-Ti, polishing substantially enhanced ductility and plateau stress of low-VED (35 and 40 J/mm^3^) samples, allowing both ultimate strength and strain at failure to exceed >2,000 MPa and >80%, respectively (Fig. 4D and Table S4). This improvement is mainly associated with the removal of residual powders and surface defects, which reduced potential sites for premature local failure. Meanwhile, the 30- to 50-μm pore enlargement caused by polishing did not substantially compromise the load-bearing framework, indicating that the optimized AO process removed surface-associated material while preserving the integrity of the main struts.

For Ti64, polished specimens displayed more pronounced dynamic stress fluctuations and multistage plateau features during yielding. At 45 J/mm^3^, polishing increased the energy absorption and strain at failure to 137.2 MJ/m^3^ and 61.05%, respectively (Fig. 4F and Table S4). Thus, although AO polishing may slightly reduce strength in some conditions, it substantially extended the plastic deformation stage and improved energy absorption, particularly at the selected 45 J/mm^3^ condition. At 50 J/mm^3^, where the printed structure already possessed relatively high densification and structural uniformity, the additional mechanical benefit of polishing was comparatively limited.

In situ deformation observations further revealed that unpolished low-VED samples exhibited premature local instability and nonuniform collapse at the onset of compression. By contrast, polishing combined with a high VED markedly delayed instability, promoted uniform deformation, and optimized stress transfer pathways. Although Ti64 lacked a stable plateau region, AO polishing mitigated surface-defect-induced failure during early loading, prolonged the plastic flow regime, and markedly improved energy absorption efficiency.

Collectively, these results demonstrate that VED primarily determines the internal structural quality and initial strength, whereas AO polishing mainly regulates surface defects, residual powder content, pore geometry, and local stress distribution. The selection of 45 J/mm^3^ was therefore based not simply on achieving the highest strength, but on maintaining sufficient structural strength while preserving deformation tolerance and energy absorption capacity. AO polishing further established a favorable balance between effective residual powder removal and limited pore enlargement while avoiding substantial deterioration of the load-bearing struts. Accordingly, CP-Ti-45 was selected as the representative condition for subsequent AO polishing and biological evaluation, providing a mechanically robust porous structure with sufficient tolerance for the surface modification induced by AO polishing.

In summary, VED predominantly governs microstructural integrity and initial strength, while AO polishing primarily enhances surface quality and stress distribution. The synergistic optimization of both parameters enables a favorable balance between strength and toughness, as further supported by the tensile stress–strain responses shown in Figs. S5 to S7 and Table S5, thereby providing comprehensive mechanical evidence for the design of customized, high-performance orthopedic implants.

### Cytocompatibility of the extract of the gyroid porous Ti sample after polishing in vitro

To systematically evaluate the effects of polishing treatment on the biological safety and osteogenesis-related cellular behaviors of 3D-printed porous Ti materials, an extract-based culture model was first employed to eliminate the interference of macroscopic structural differences. This approach allowed focused investigation of the indirect biological effects induced by changes in surface state, including residual powder removal as well as alterations in surface chemistry and microstructure. By comparing different material systems (CP-Ti and Ti64) and surface conditions, the influence of polishing treatment on osteoblast apoptosis, proliferation, and migration was systematically analyzed.

The cell apoptosis rate was assessed using annexin V–fluorescein isothiocyanate/propidium iodide double staining followed by flow cytometry (Fig. 5A). Compared with the control group, preosteoblasts cultured in extracts from porous materials exhibited significantly decreased proportions of early and late apoptotic cells. Meanwhile, cells cultured in extracts from AO-polished porous materials showed a slightly reduced apoptosis rate than the none AO-polished material. Among the 2 material systems, both CP-Ti-AU-45 and Ti64-AU-45 groups demonstrated lower levels of apoptosis, with the CP-Ti-AU-45 group exhibiting a more pronounced anti-apoptotic effect.

**Fig. 5.**
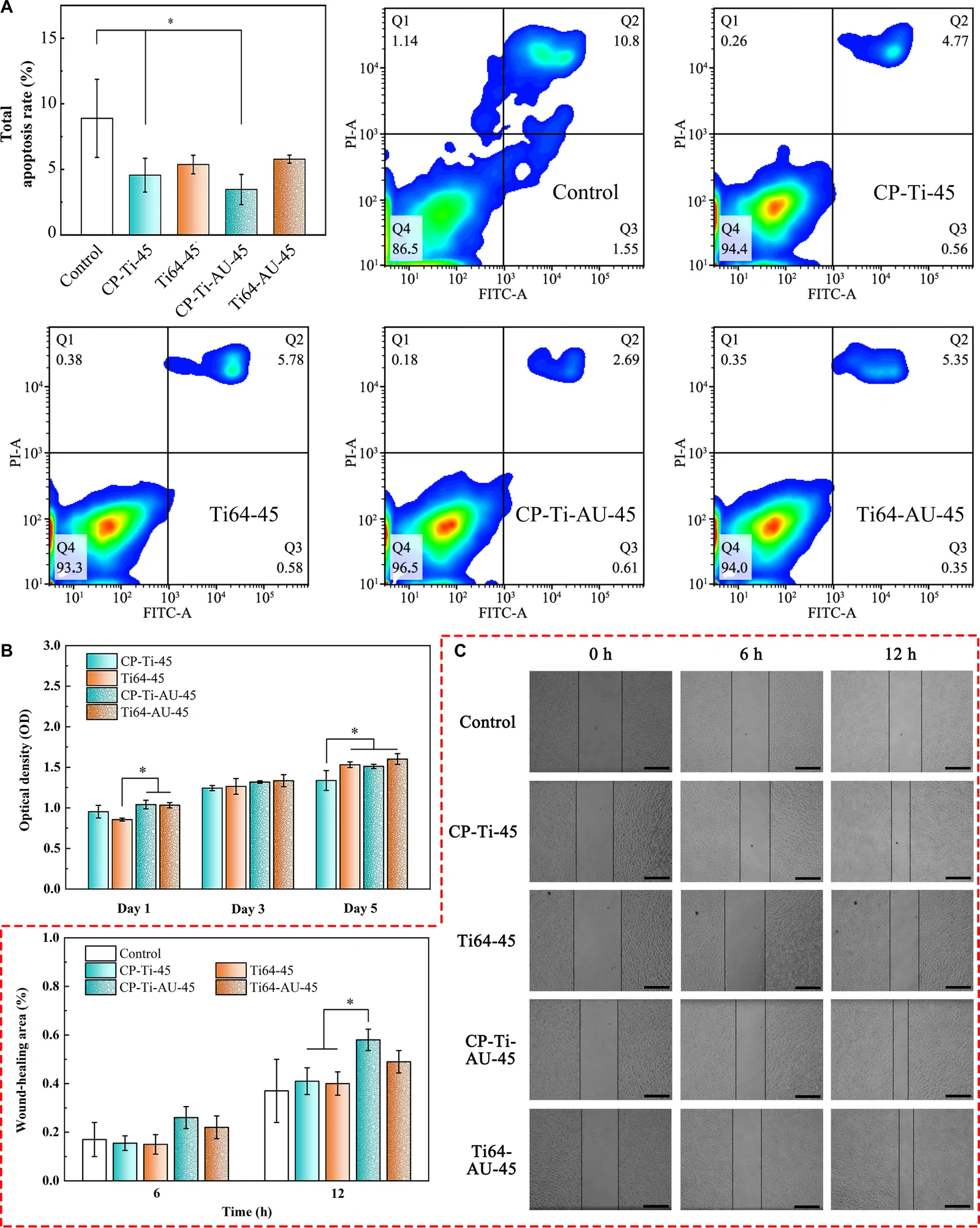
(A) Apoptosis rate of MC3T3-E1 cells cultured in the extract of gyroid porous CP-Ti-45, Ti64, CP-Ti-AU-45, and Ti64-AU-45 samples (*P < 0.05). (B) Cytotoxicity and (C) wound-healing area of MC3T3-E1 cells cultured in the extract of gyroid porous CP-Ti-45, Ti64-45, CP-Ti-AU-45, and Ti64-AU-45 samples (scale bar = 500 μm, *P < 0.05).

Cell proliferation under different extract conditions was evaluated using the CCK-8 assay at 1, 3, and 5 d (Fig. 5B). All groups exhibited a time-dependent increase in cell proliferation during the culture period. Notably, at days 3 and 5, osteoblast proliferation in the CP-Ti-AU-45 and Ti64-AU-45 groups was significantly higher than that in the corresponding unpolished groups and exceeded that of the control group. These results indicate that AO-polished porous materials not only exhibited no inhibitory effects on osteoblast proliferation but instead significantly promoted cell growth.

The effects of material extracts on osteoblast migration were further evaluated using a scratch wound-healing assay (Fig. 5C). With increasing culture time, progressive closure of the scratch area was observed in all groups. Compared with unpolished groups, AO-polished groups exhibited significantly higher wound closure rates at both 6 and 12 h. Among them, the CP-Ti-AU-45 group showed a more rapid scratch closure, with the migrated area at 12 h being significantly greater than that of the control and unpolished groups. These results demonstrate that extracts from polished porous materials markedly enhance osteoblast migratory capacity, which is beneficial for early cell recruitment and surface coverage on implant interfaces.

Taken together, the improved cellular behaviors observed in this study indicate that polishing treatment significantly enhances the cytocompatibility of porous metal materials by effectively removing residual powders and modulating surface chemical states and microstructural features. Suppression of osteoblast apoptosis may represent a critical prerequisite mechanism by which AO-polished porous materials promote cell proliferation and migration, thereby providing a favorable cellular basis for subsequent osteogenic differentiation and osseointegration.

### In vitro biocompatibility of polished porous Ti metals

The initial adhesion of osteoblasts to implant surfaces is a critical indicator of the biocompatibility of biomaterials and their potential for early osseointegration [33]. Robust early adhesion often predicts subsequent cell proliferation, differentiation, and the establishment of a stable bone–implant interface [34].

In this study, the early adhesion behavior of osteoblasts on porous metals was examined (Fig. 6A). SEM observations revealed that unpolished samples (CP-Ti-45 and Ti64-45) retained residual powder particles on the surface, yet the cells exhibited a flattened morphology with abundant filopodia, indicating basic adhesive capacity. In contrast, after high-voltage AO polishing (CP-Ti-AU-45 and Ti64-AU-45), cells displayed a more spread and plump morphology, with filopodia extending along honeycomb-like pits and micro/nanostructures, suggesting a more stable attachment. These findings demonstrate that the polished surface topography facilitates cellular adhesion and spreading.

**Fig. 6.**
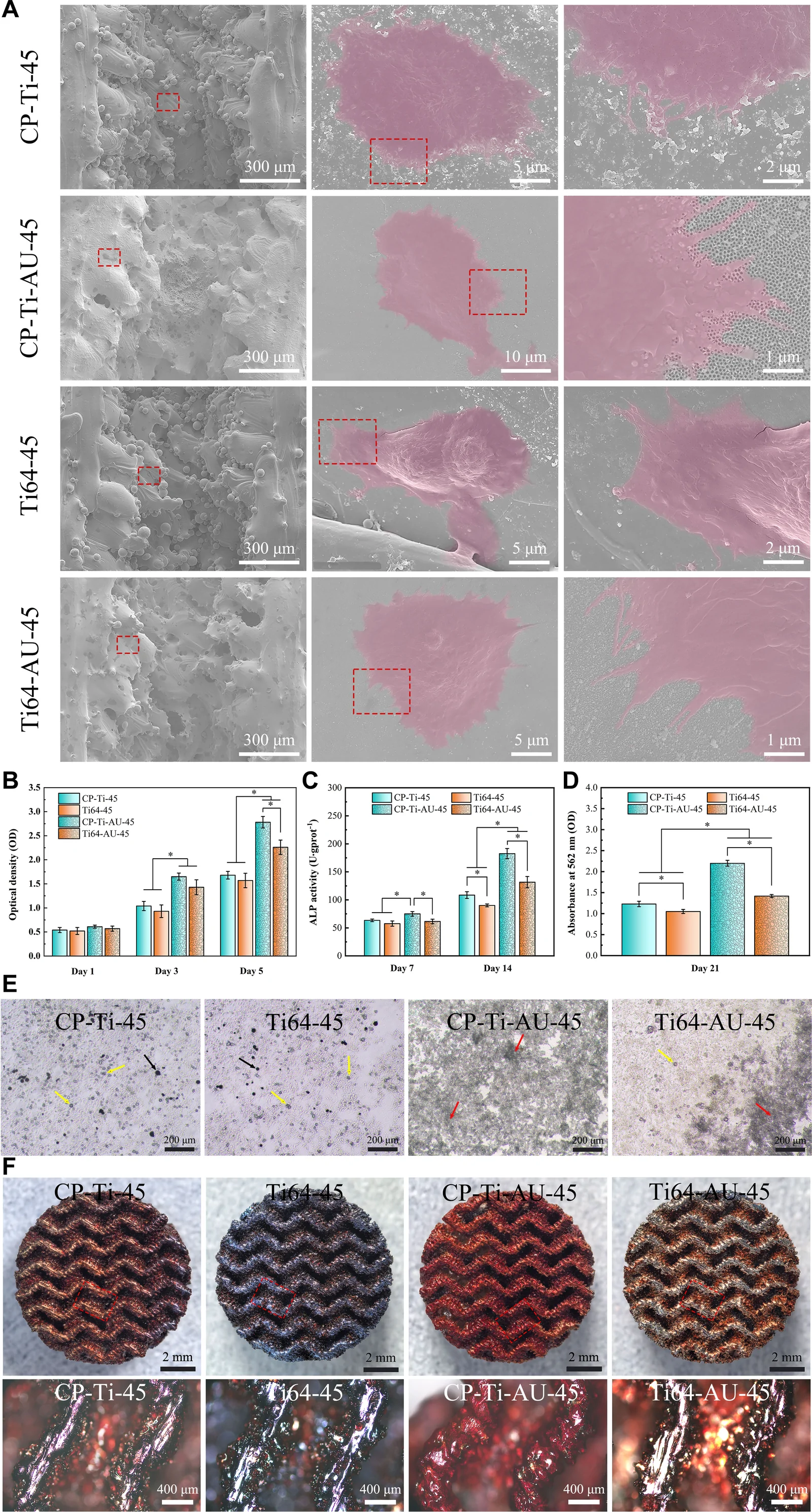
(A) SEM images of MC3T3-E1 cells cultured on gyroid porous CP-Ti-45, CP-Ti-AU-45, Ti64-45, and Ti64-AU-45 samples for 2 h. (B) Cytotoxicity of MC3T3-E1 cells cultured on gyroid porous CP-Ti-45, Ti64-45, CP-Ti-AU-45, and Ti64-AU-45 samples for 1, 3, and 5 d (*P < 0.05). (C) Alkaline phosphatase (ALP) activities of gyroid porous samples after incubation for 7 and 14 d. (D) Quantification of calcium deposition on various sample surfaces (*P < 0.05). (E) Optical morphology of MC3T3-E1 cells surrounding gyroid porous CP-Ti-45, Ti64-45, CP-Ti-AU-45, and Ti64-AU-45 samples after incubation for 14 d; residual powder (black arrows) dropped from the samples, and small granular (yellow arrows) or sheetlike mineralized calcium nodules (red arrows) were observed. (F) Low- and high-magnification images of the extracellular matrix (ECM) mineralization of MC3T3-E1 cells after incubation for 21 d with gyroid porous CP-Ti-45, Ti64-45, CP-Ti-AU-45, and Ti64-AU-45 samples in vitro.

In proliferation assays, all samples showed a progressive increase in optical density (OD) values from day 1 to day 5, confirming good cell viability and compatibility (Fig. 6B). Notably, polished samples exhibited significantly higher OD values at days 3 and 5 compared with unpolished counterparts. Among them, CP-Ti-AU-45 reached 2.78 ± 0.12 at day 5, markedly higher than those of CP-Ti-45 (1.68 ± 0.08) and the Ti64 series. Furthermore, direct comparison under polished conditions indicated that CP-Ti-AU-45 supported superior cell proliferation relative to Ti64-AU-45 (2.28 ± 0.15), underscoring the favorable influence of its substrate properties and surface structure on osteoblast growth.

Osteogenic differentiation, a key determinant of bone integration, was subsequently assessed. By day 14, cells cultured on porous metals exhibited enhanced osteogenic activity, with both surface-adherent and peripheral MC3T3-E1 cells displaying mineralization. Large calcium nodules were observed around Ti64-AU-45, while CP-Ti-AU-45 showed extensive sheetlike nodules formed by fused cell aggregates, indicative of more pronounced and mature mineralization (Fig. 6E).

ALP activity, an early marker of osteogenic differentiation and ECM maturation, increased significantly in all groups by day 14 [35]. The CP-Ti-AU-45 group showed the highest ALP activity (182.49 ± 9.08 U·gprot^−1^), surpassing all other groups, which was consistent with the optical microscopy findings (Fig. 6C). This enhancement may be attributed to the effective removal of residual powders, the stable micro/nanostructures, and the favorable surface chemistry of CP-Ti-AU-45.

ECM mineralization, a hallmark of late-stage osteogenesis, was further examined by ARS staining at day 21 [36]. Deep red regions indicating calcium deposition were observed on all samples, with CP-Ti-AU-45 exhibiting extensive and intense staining (Fig. 6F). Quantitative analysis confirmed that its OD value (2.19 ± 0.07) was ~1.5-fold higher than that of Ti64-AU-45 (Fig. 6D). High-magnification SEM revealed abundant mineralized nodules tightly anchored to the honeycomb-like and ridgelike micro/nanostructures, highlighting their role in providing anchoring sites and interfaces conducive to protein adsorption and matrix mineralization.

Taken together, high-voltage AO polishing effectively removed surface contaminants and generated ordered honeycomb-like and multiscale micro/nanostructures on porous metals, thereby markedly promoting osteoblast adhesion, sustained proliferation, and subsequent differentiation and mineralization. Among all groups, CP-Ti-AU-45 consistently exhibited the most favorable outcomes, demonstrating superior in vitro osteogenic performance.

To investigate the regulatory effects of high-voltage AO polishing on osteogenic-differentiation-related signaling pathways, transcriptomic analysis was performed on cells cultured on CP-Ti-AU-45 and Ti64-AU-45 surfaces. DEGs were identified using FC > 1.5 and FDR < 0.05 as the screening criteria. Both materials induced pronounced transcriptional alterations, with the number of up-regulated genes markedly exceeding that of down-regulated genes, indicating that AO-polished surfaces effectively activated pathways associated with cellular metabolism and differentiation.

In the CP-Ti-AU-45 group, DEGs were predominantly enriched in classical osteogenic signaling pathways, including PI3K–Akt, mitogen-activated protein kinase (MAPK), and transforming growth factor-β (TGF-β). Key osteogenesis-related genes, such as Col1a1, Alpl, Runx2, and Bmp2, were significantly up-regulated (*P* < 0.05), demonstrating that AO-polished CP-Ti surfaces effectively promoted the activation of osteogenic signaling (Fig. 7A). Combined with surface characterization results, these findings suggest that AO polishing reduced residual powder, optimized surface micro/nanostructures, and improved surface energy, thereby enhancing cell adhesion and signal integration to create a favorable microenvironment for osteogenic differentiation [37].

**Fig. 7.**
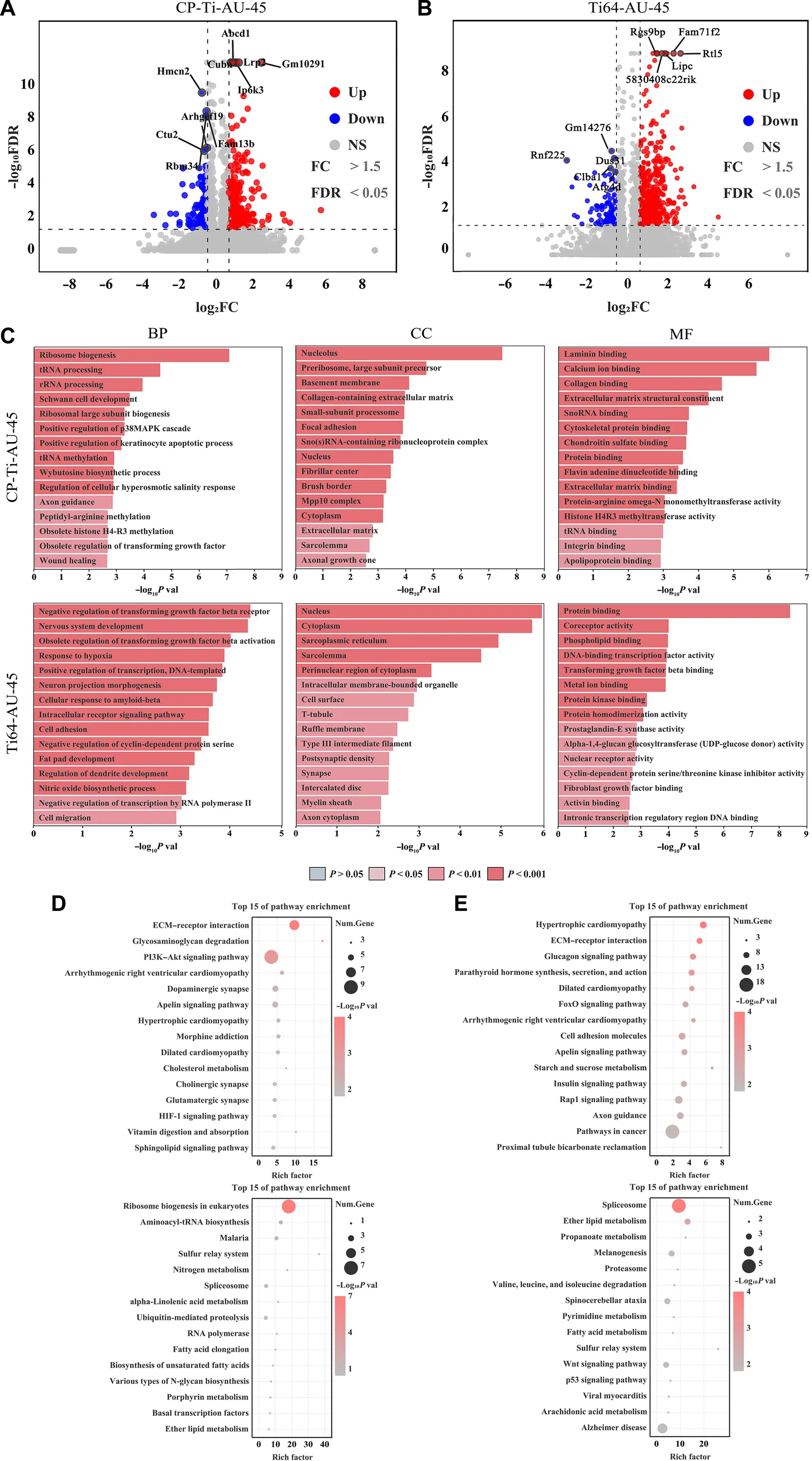
Volcano plots of differentially expressed genes in MC3T3-E1 cells after incubation for 3 d with polished gyroid porous (A) CP-Ti-AU-45 and (B) Ti64-AU-45 samples in vitro. (C) Gene Ontology (GO) enrichment analysis of differentially expressed genes in MC3T3-E1 cells after incubation for 3 d with polished gyroid porous CP-Ti-AU-45 and Ti64-AU-45 samples. Kyoto Encyclopedia of Genes and Genomes (KEGG) pathway enrichment analysis of DEGs in MC3T3-E1 cells after 3 d of incubation with polished gyroid porous (D) CP-Ti and (E) Ti64 samples, showing the top 15 enriched pathways for up-regulated genes and down-regulated genes.

In contrast, the Ti64-AU-45 group also exhibited a predominant up-regulation trend; however, the enriched pathways were mainly associated with mechanosensing and cytoskeletal remodeling, such as Wnt/β-catenin and integrin–focal adhesion kinase (FAK) signaling pathways (Fig. 7B). This indicates that AO-polished Ti64 surfaces primarily promote osteogenic phenotypes through the modulation of cell adhesion structures and mechanotransduction processes.

Further GO and Kyoto Encyclopedia of Genes and Genomes (KEGG) enrichment analyses were conducted to elucidate the underlying molecular mechanisms. GO functional enrichment analysis revealed that cells cultured on both CP-Ti-AU-45 and Ti64-AU-45 surfaces exhibited pronounced enrichment trends at the levels of biological process, cellular component, and molecular function, indicating that AO-polished surfaces modulated cellular behaviors in a multi-level manner (Fig. 7C). GO analysis revealed that DEGs in the CP-Ti-AU-45 group were mainly enriched in biological processes related to cell adhesion, ECM organization, and osteoblast differentiation, whereas the Ti64-AU-45 group showed enrichment in cell migration, cytoskeleton organization, and signal transduction regulation. In the CP-Ti-AU-45 group, DEGs were predominantly enriched in canonical osteogenic signaling pathways, including PI3K–Akt, MAPK, TGF-β, focal adhesion, and ECM–receptor interaction pathways (Fig. 7D). Key osteogenic regulators, such as Col1a1, Bmp2, and Runx2, were significantly up-regulated, indicating that AO-polished surfaces may activate PI3K–Akt and MAPK signaling cascades and thereby promote osteogenesis-related gene expression and ECM formation.

By contrast, the Ti64-AU-45 group exhibited activation of a more limited set of pathways mainly related to mechanotransduction, angiogenesis, and immune regulation. Although the overall regulatory trend was consistent with that observed in CP-Ti-AU-45, the magnitude of pathway enrichment and gene up-regulation was comparatively weaker (Fig. 7E). KEGG analysis further confirmed that the PI3K–Akt pathway was significantly activated in the CP-Ti-AU-45 group, serving as a central regulatory axis for AO polishing-induced osteogenic differentiation (Fig. S8).

To further elucidate the molecular mechanisms underlying the enhanced osteogenic performance of Ti64-AU-45, a comparative transcriptomic analysis was conducted between the Ti64-AU-45 and Ti64-45 groups. The volcano plot (Fig. S9A) revealed significant up-regulation of key osteogenic and adhesive markers, including Runx2, Smad9, Tgfbi, Mef2c, and Pdgfra, in the Ti64-AU-45 group compared with the Ti64-45 group, while genes such as Sf3b4, Dpp7, Slc10a3, Adal, and Lpcat3 were significantly down-regulated. Protein–protein interaction network analysis (Fig. S9B) demonstrated a highly interconnected cluster comprising Runx2, Smad9, Pdgfra, and Mef2c, suggesting that these interconnected factors may collectively contribute to the osteogenic response following AO. Additionally, the clustering of Itga2, Itgb8, Tgfbi, and Thbs1 suggests that the surface micro-nano topography may facilitate initial cell adhesion and migration through these molecular mediators.

Functional enrichment via KEGG pathway analysis (Fig. S9C) showed that the DEGs were predominantly enriched in ECM–receptor interaction, focal adhesion, and the PI3K–Akt signaling pathway, which are critical for mediating cell–material cross talk. The results also demonstrated a relative down-regulation of ferroptosis-related signaling pathways. These findings were further corroborated by the quantitative expression levels of representative DEGs (Fig. S9D), where the messenger RNA levels of Runx2 and Mef2c, master regulators of osteogenesis, exhibited a marked increase post-AO. Furthermore, GO analysis (Fig. S9E) highlighted the significant enrichment of integrin binding and the positive regulation of ALP activity.

Collectively, these results suggest that AO provides a bioactive interface that may promote osteoblast differentiation through integrin-associated mechanotransduction and downstream osteogenic signaling cascades. The observed down-regulation of Lpcat3 and ferroptosis-related pathways further suggests that modulation of ferroptosis-related signaling may be involved in the improved cellular response, although this interpretation requires further protein-level and functional validation.

### In vivo osteogenic activity of polished porous Ti metals

At 4 weeks postimplantation, micro-CT reconstruction revealed evident powder detachment and insufficient new bone formation around the unpolished porous implants, with detached particulate residual powders observed around the struts, suggesting that residual surface particles may impair bone integration (Fig. 8A and B). In contrast, the high-voltage AO-polished samples exhibited markedly reduced residual powder and abundant bone ingrowth along the pore walls and inner surfaces, indicating improved bone extension and climbing capability. Notably, in the CP-Ti-AU-45 specimens, new bone grew densely along the pore walls, filling the internal structures, narrowing trabecular spacing, and forming intimate contact with the implant surface. BIC reached 61.7%, approaching the physiological level reported for the natural tooth root–alveolar bone interface (80% to 85%) (Fig. 8C). These findings demonstrate the excellent osseointegration capacity of the CP-Ti-AU-45 implant at the microscale. Quantitative analysis further confirmed this observation: CP-Ti-AU-45 achieved the highest BMD, BV/TV, Tb.N, and Tb.Th values (0.19 g/cm^3^, 26.7%, 7.4 1/mm, and 0.23 mm, respectively) while showing the lowest Tb.Sp (0.17 mm) (Fig. 8D to H).

**Fig. 8.**
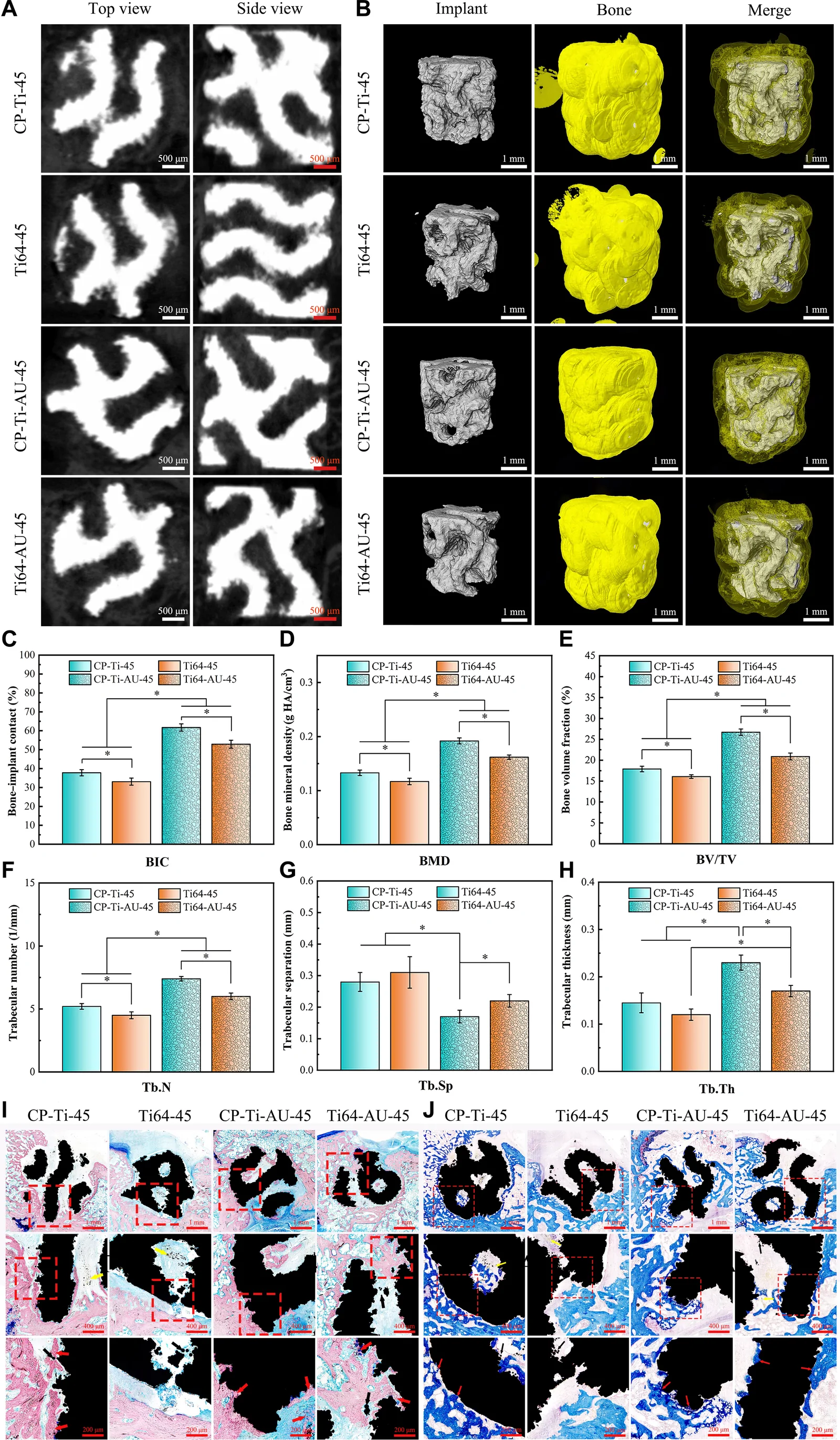
(A) Representative original micro-computed tomography (micro-CT) slice images showing the top and side views of gyroid porous Ti implants. (B) Micro-CT reconstruction images of gyroid porous Ti implants and surrounding newly formed bone after implantation for 4 weeks. Quantitative analysis of osteogenic parameters, namely, (C) bone–implant contact (BIC), (D) bone mineral density (BMD), (E) bone volume fraction (BV/TV), (F) trabecular number (Tb.N), (G) trabecular spacing (Tb.Sp), and (H) trabecular thickness (Tb.Th) around gyroid porous implants after implantation for 4 weeks (*P < 0.05). (I) Methylene blue and (J) toluidine blue staining of new bone around polished gyroid porous Ti implants after implantation for 4 weeks. Panoramic, low-magnification, and high-magnification images are presented from top to bottom, showing detached residual powders on the inner and outer surfaces of the pores (black arrows) and the associated immune responses (yellow arrows), as well as a serrated interface between the newly formed bone and the implant (red arrows).

Methylene blue staining revealed consistent results. In the unpolished groups, a thick fibrous capsule and residual particles were frequently observed at the implant–bone interface, accompanied by localized inflammatory responses (Fig. 8I). Notably, detached particles were also observed within the surrounding tissue, particularly near regions with evident particle aggregation (Fig. S10A). Inflammatory cell infiltration and fibrous tissue proliferation were particularly evident in regions with particle aggregation (5 to 30 μm), presenting typical morphological features of inflammation and indicating interference with bone integration. These histological findings were consistent with the micro-CT observations and provided direct evidence of in vivo powder detachment from the untreated porous implants. In contrast, the polished groups exhibited “sawtooth-like” interfacial bonding, with trabeculae extending along pore walls to form a 3D bone network. Importantly, no substantial accumulation of fine powder or soft tissue was observed either inside the pores or at the periphery of polished implants, confirming that AO polishing efficiently removed loose and weakly adhered particles, thereby improving the interfacial fusion and biocompatibility. Particularly in CP-Ti-AU-45, a fibrocartilage-to-lamellar bone transition was detected within the pores, indicating continuous osteoinductive activity. Toluidine blue staining further validated the bone ingrowth behavior and was in strong agreement with the methylene blue results (Fig. 8J and Fig. S10B).

To comprehensively assess bone mineralization and osseointegration, VG staining was performed (Fig. 9A). Mature bone appeared red; newly formed bone, orange-red; osteoid, deep yellow; and soft tissue, light yellow. In unpolished groups, residual-particle-associated inflammatory responses were again observed, together with localized fibrous tissue formation and reduced bone formation in particle-rich regions (Fig. 9B). Notably, in the CP-Ti-45 group, the interface bone tissue stained dark orange-red, suggesting a higher degree of mineralization compared with other groups. In polished groups, VG staining displayed results consistent with those from methylene blue and toluidine blue. Remarkably, extensive deposition of osteoid (deep yellow) was observed at both the interface and within pores, particularly in CP-Ti-AU-45, where osteoid covered a significantly larger area than in other groups and formed distinct transition zones with new bone, suggesting enhanced osteoinductive and bone-mineralization regulatory capacity.

**Fig. 9.**
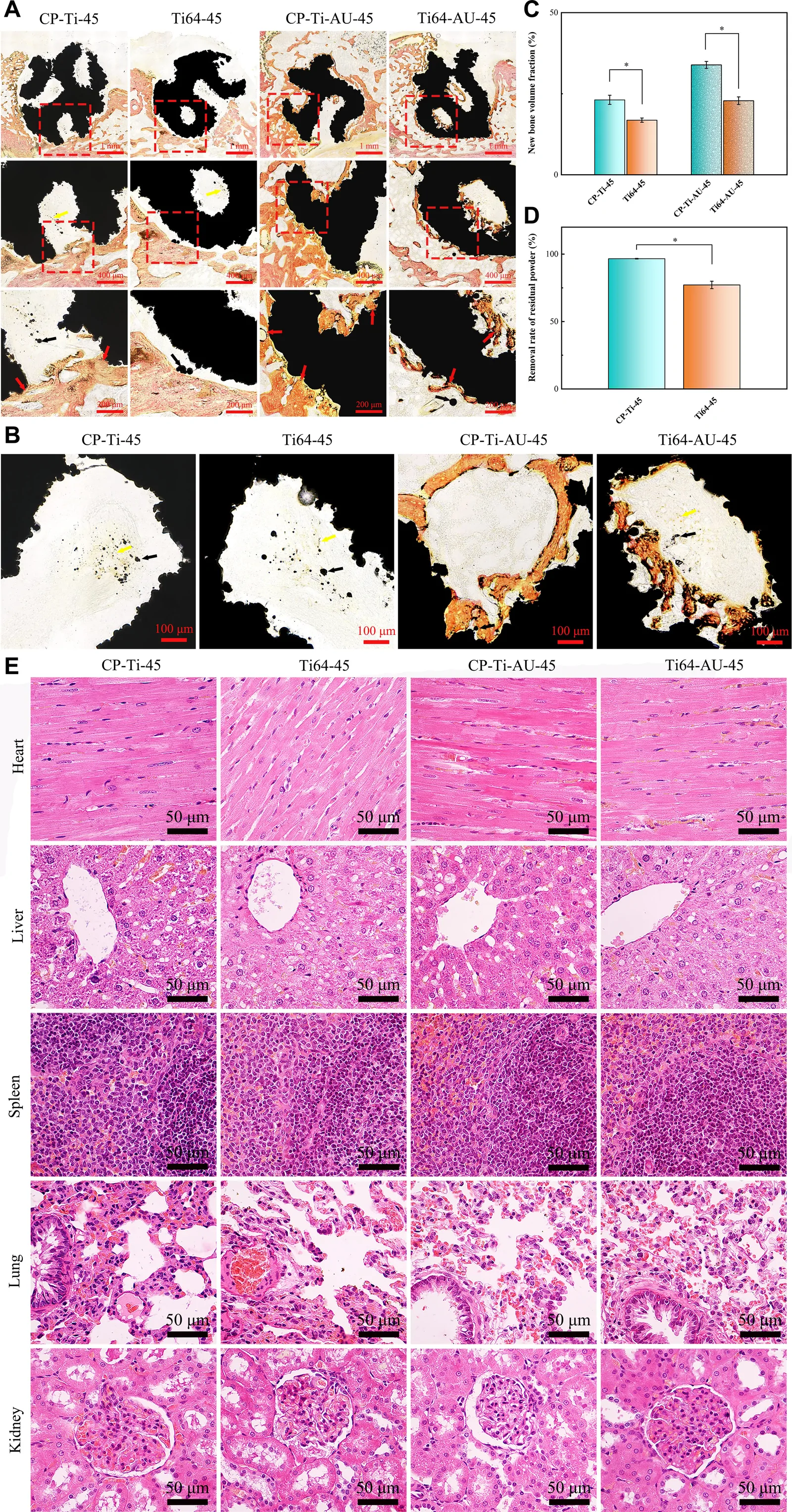
(A) Van Gieson staining of new bone around polished gyroid porous Ti implants after implantation for 4 weeks. Panoramic, low-magnification, and high-magnification images are presented from top to bottom, showing detached residual powders on the inner and outer surfaces of the pores (black arrows) and the associated immune responses (yellow arrows), as well as a serrated interface between the newly formed bone and the implant (red arrows). (B) Enlarged images of selected regions showing residual powders in Van Gieson-stained sections. Quantitative analysis of (C) newly formed bone and (D) residual powders around the gyroid porous Ti implants after implantation for 4 weeks. (E) H&E staining of the rat heart, liver, spleen, lung, and kidney tissues from polished gyroid porous Ti implants after implantation for 4 weeks.

VG-stained sections were quantitatively analyzed to evaluate new bone formation. Within a 250-μm peri-implant zone, the new bone volume fractions in CP-Ti-45 and Ti64-45 were (23.1 ± 1.4)% and (16.8 ± 0.7)%, respectively. After AO polishing, the values increased to (33.8 ± 1.1)% in CP-Ti-AU-45 and (22.8 ± 1.2)% in Ti64-AU-45 (Fig. 9C). Statistical analysis confirmed that AO polishing significantly promoted peri-implant new bone formation, with CP-Ti-AU-45 exhibiting the most pronounced effect.

Residual powder removal was quantitatively assessed based on VG-stained sections using the ImageJ software. The removal efficiencies for CP-Ti-45 and Ti64-45 implants were 96.6% ± 0.2% and 77.1% ± 2.8%, respectively (Fig. 9D). These findings are consistent with histological observations, following the trend of “reduced powder → attenuated inflammation → enhanced bone integration”, and further verify the superior cleaning efficiency of AO polishing in complex porous structures, especially in CP-Ti samples.

At week 4, major organs (heart, liver, spleen, lung, and kidney) were harvested and examined histologically by H&E staining. As shown in Fig. 9E, no evident pathological abnormalities, including tissue disorganization, cellular degeneration, necrosis, hemorrhage, or inflammatory infiltration, were detected in either polished or unpolished groups, suggesting the favorable systemic biocompatibility of the implants.

Collectively, in vivo analyses demonstrated that high-voltage AO polishing effectively removed residual powders, improved the interfacial microenvironment, and significantly promoted new bone formation and osseointegration. The combined micro-CT and histological observations further demonstrated that residual powders in the untreated implants could detach in vivo and were associated with localized inflammatory and fibrotic responses, whereas these adverse features were substantially reduced after AO polishing. Among all tested groups, CP-Ti-AU-45 exhibited the best osteogenic performance and powder removal efficiency, underscoring its potential clinical advantages as a porous metallic implant.

## Discussion

### Removal mechanism of residual powders from porous Ti metals during polishing

High-voltage AO polishing refers to a surface treatment process in which titanium or its alloys are subjected to controlled electrochemical conditions, where a high voltage is applied in an electrolyte containing fluoride ions. Under these conditions, an ordered yet mechanically fragile TiO_2_ nanotubular array is formed on the metal surface. The subsequent application of low-frequency ultrasonication disrupts this unstable structure, thereby enabling the effective removal of residual powders embedded in the deep porous regions while simultaneously generating new, stable micro/nanostructured surface patterns. A schematic illustration of the high-voltage AO polishing process for CP-Ti is shown in Fig. 10, and the main stages can be summarized as follows:

1. Barrier layer formation: When the titanium substrate is used as the anode in the electrochemical cell, a dense TiO_2_ barrier layer is first formed at the metal–electrolyte interface under a high voltage (Fig. 10B). The thickness of this barrier layer depends on both the applied voltage and oxidation time. Simultaneously, in the oxidative environment of the electrolyte, mechanical stirring promotes the electrochemical decomposition of water, where Ti ions react with oxygen or hydroxyl species to form the initial oxide layer [38], as described by the following reaction:

$$\mathrm{Ti}+2H_{2}O\to \mathrm{Ti}O_{2}+4H^{+}+4e^{-}$$ (3)

2. Nucleation and growth of nanotubes: With increasing voltage, localized breakdown occurs in the barrier layer due to chemical attack by F^−^ and other anions in the electrolyte, resulting in nanoscale pore formation [39]. The electric field is locally intensified at these pores, accelerating their further breakdown and leading to the formation of tubular structures that progressively extend into the oxide layer (Fig. 10C). Metal ions migrate through the oxide layer and react to form soluble titanium fluoride complexes, as shown in the following reaction [40]:

$$\mathrm{Ti}O_{2}+4H^{+}+6F^{-}\to {\left[\left.\mathrm{Ti}F_{6}\right]\right.}^{-}+2H_{2}O$$ (4)

**Fig. 10.**
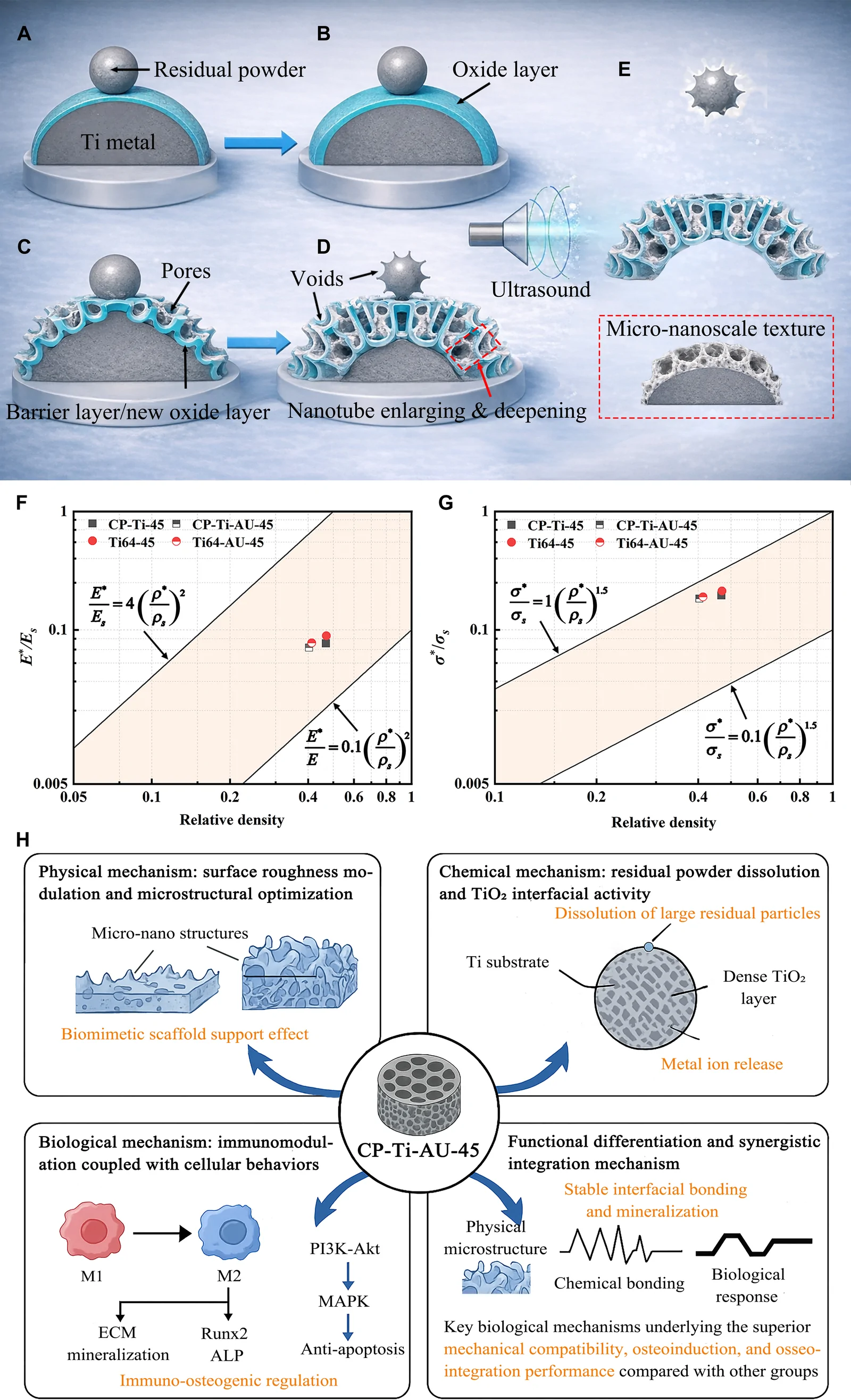
(A to E) Schematic diagram of micro-nanoscale texture formation on the surface of gyroid porous CP-Ti-AU-45 samples after high-voltage anodic oxidation (AO) polishing. (F) Relative elastic modulus and (G) relative density of gyroid porous CP-Ti-45, CP-Ti-AU-45, Ti64-45, and Ti64-AU-45 samples before and after polishing. (H) Schematic illustration of the multi-level mechanisms underlying bone regeneration induced by CP-Ti-AU-45 samples.

The growth of nanotubes is governed by a dynamic balance between the field-assisted oxidation reaction and the dissolution process. Under this balance, TiO_2_ is continuously generated at the tube base while the tube tips undergo dissolution under the influence of F^−^ and H^+^, producing vertically aligned nanotubular arrays.

3. Dynamic evolution of nanotube structures: When the applied voltage and electrolyte temperature (∼40 °C) are maintained within a suitable range, the growth rate of oxide at the oxide/metal interface becomes lower than the dissolution rate of oxide at the oxide/electrolyte interface. Under such conditions, nanotube arrays develop with increasing tube length and diameter, accompanied by progressive thinning of the tube walls (Fig. 10D). The high-voltage environment sustains the inward growth of the oxide layer and facilitates the formation of well-defined nanotube structures.
4. Nanotube detachment and powder removal: After approximately 50 min of high-voltage anodization, the nanotubes reach a critical length, and the walls become sufficiently thin such that accumulated mechanical and chemical stresses promote localized detachment of tube bundles. Low-frequency ultrasonication further facilitates the removal of these fragile nanotube segments as well as loosely attached residual powders from both the surface and the porous interior (Fig. 10E).

This AO-assisted polishing strategy demonstrates excellent effectiveness for removing residual powders in porous CP-Ti and Ti64 samples, with CP-Ti exhibiting particularly stable and reproducible results. Ultrasonication not only exposes robust nanotube roots but also eliminates loose particles, ultimately generating favorable micro/nanostructures such as honeycomb-like arrays, serrated micropits, and spine- or Haversian canal-like patterns. These features provide topographies that are highly conducive to cellular adhesion and growth. Importantly, the diameter, length, wall thickness, and structural stability of the nanotubes are critically determined by voltage, temperature, processing time, and electrolyte composition.

Under the optimized anodization parameters employed in this study, uniform and well-controlled nanotubular structures could be stably produced on CP-Ti surfaces. By contrast, Ti64 samples showed relatively poor structural stability, with localized detachment of nanotubes occurring more frequently during anodization. Although ultrasonic treatment retained a fraction of the micro/nanostructures, the degree of ordering and integrity was markedly inferior to that of CP-Ti. Future optimization of both the 3D-printing parameters and the AO polishing conditions is expected to improve the uniformity and robustness of nanotube arrays on Ti64, thereby enabling biologically functional surface modifications for orthopedic applications.

In summary, high-voltage AO polishing not only elucidates the mechanism underlying the removal of residual powders in porous Ti implants but also clarifies the formation of bioinspired micro/nanostructures, thereby providing a mechanistic foundation for the subsequent optimization of their mechanical and biological performance.

### Change in the mechanical properties of porous Ti metals after polishing

Under identical laser energy density (VED) conditions, high-voltage AO polishing markedly enhanced the compressive performance of porous Ti alloys. Taking CP-Ti-AU specimens fabricated at low VEDs (35 and 40 J/mm^3^) as an example, the ultimate compressive strength exceeded 2,000 MPa, while the strain to failure increased beyond 80%, demonstrating superior ductility and energy absorption capacity. Compared with the as-printed samples, this improvement can be attributed to the synergistic contributions of the following 3 factors:

• Surface state optimization: During AO polishing, residual powders and localized oxide films were effectively removed, leading to an average pore enlargement of ~30 to 50 μm without causing substantial damage to the structural integrity of the pore walls. Consequently, the pore surfaces became more intact, free of loosely adhered particles that could act as crack initiators. Moreover, the reduction in surface roughness mitigated stress concentration, enabling more homogeneous stress transfer and distribution at the early stages of deformation, thereby delaying localized instability and collapse.
• Release of internal stresses: The combined effect of AO polishing with low-frequency ultrasonic agitation efficiently relieved residual thermal stresses generated during the printing process and facilitated stress redistribution. This reduction in internal stresses enhanced load stability during compression and improved structural integrity, allowing the material to retain higher toughness and energy dissipation capacity under large deformations.
• Porosity and modulus regulation: Although polishing increased the average porosity of the samples by ~3% to 6% and slightly reduced their relative density (e.g., from 0.195 to 0.183 for CP-Ti-AU-45), the decreases in elastic modulus and yield strength were limited to only 0.1% to 3%, much smaller than the values predicted solely from porosity increase. This indicates that the thinning of pore walls induced by AO treatment was more uniform, avoiding irregular defects that would otherwise undermine stability. Importantly, the elastic modulus of 3 to 10 GPa closely matches that of natural cancellous bone, thereby reducing stress shielding and providing a mechanically compatible environment for bone ingrowth.

From the macroscopic perspective, unpolished samples fabricated at a low VED exhibited early onset of localized strain concentration and instability, manifesting as collapse or twisting. In contrast, polished samples displayed more uniform stress transfer pathways and delayed local failure, which substantially enhanced energy dissipation efficiency. In other words, the combined optimization of surface integrity and internal stress states endowed porous CP-Ti scaffolds with greater structural stability, enabling sustained load-bearing capacity under large compressive strains.

To further evaluate the coupling relationship between high-voltage AO polishing treatment and the structural–mechanical performance of gyroid porous metals, representative samples with superior compressive properties, including CP-Ti-45, CP-Ti-AU-45, Ti64-45, and Ti64-AU-45, were selected for analysis. The experimental mechanical data were interpreted using the Gibson–Ashby model, which describes the mechanical behavior of porous metals as a function of relative density [41,42].

According to this model, the relative elastic modulus (E^*^/*E_s_*) and relative yield strength (σ^*^/*σ_s_*) of porous structures scale with the relative density (ρ^*^/*ρ_s_*) following power-law relationships. The constants *C*_1_ and *C*_2_ and exponents *n*_1_ and *n*_2_ are geometry-related fitting parameters. For dense CP-Ti, the values of *E_s_*, *σ_s_*, and *ρ_s_* were taken as 110 GPa, 533 MPa, and 4.506 g·cm^−3^, respectively [43], whereas those for dense Ti64 were set to 110 GPa, 1,015 MPa, and 4.43 g·cm^−3^, respectively, based on reported literature values [44].

Taking CP-Ti-45 and CP-Ti-AU-45 as representative examples, the porosity increased from approximately 53.2% to 59.7% after polishing treatment, corresponding to a reduction in relative density of about 3% to 6%. Despite this change, only slight decreases in the elastic modulus and yield strength were observed (approximately 0.1% to 3%), indicating that the porous structure maintained good mechanical stability even under conditions of reduced strut thickness (Fig. 10F and G and Table S6).

Corroborated by Gibson–Ashby model fitting, the mechanical evolution of polished samples adhered closely to the classical power-law relationships (elastic modulus exponent *n*_1_ ≈ 2.1 and yield strength exponent *n*_2_ ≈ 1.5), confirming the predictable influence of porosity and pore wall thickness on mechanical performance. Further nTop simulations showed that AO polishing reduced the average pore wall thickness by ~30 to 50 μm, leading to a 3% to 5% increase in porosity, which was consistent with the experimental measurements. These findings indicate that AO polishing not only improved the actual mechanical response by removing defects but also preserved the structural regularity that underpins the predictability of mechanical behavior.

In summary, high-voltage AO polishing enhanced the ductility, energy absorption, and load-bearing stability of porous Ti alloys through a multifaceted mechanism of “defect and stress removal–pore wall morphology optimization–porosity and modulus regulation”. This integrated improvement not only mitigated the mechanical degradation typically associated with increased porosity but also brought the elastic response closer to that of natural bone, thereby substantially strengthening the clinical potential and reliability of these porous Ti scaffolds for orthopedic applications.

### Bone regeneration mechanisms of porous Ti metals after polishing

In vivo experiments based on a rat femoral defect model confirmed that high-voltage AO polishing markedly enhanced the osteogenic potential and osseointegration of porous Ti implants. These findings can be rationalized by disentangling the physical, chemical, and biological mechanisms and by distinguishing the adverse effects of residual powders from the beneficial influence of bioinspired micro/nanostructures.

Surface topography and roughness are decisive physical cues for cell adhesion and bone ingrowth. Untreated porous implants often retain a large amount of loosely bonded unmelted powders, which may transiently promote adhesion due to increased roughness but readily detach in vivo, triggering local inflammation and fibrous encapsulation. AO polishing effectively reduced surface roughness (Ra decreased from 1.44 to 1.07 μm for CP-Ti) while retaining hierarchical micro/nanostructures, such as honeycomb-like pores, serrated pits, and Haversian canal-like channels. These features provide stable anchoring sites, support cytoskeletal remodeling, and create a biomimetic scaffold interface that favors early bone apposition [34,45]. Moreover, the overall pore size remained within the 300- to 600-μm range favorable for tissue ingrowth and vascularization despite the approximately 30- to 50-μm pore-size enlargement induced by AO polishing while mitigating stress shielding and establishing a mechanically and biologically favorable niche.

Residual metallic powders can release toxic ions (e.g., Al^3+^ and V^5+^), inducing excessive reactive oxygen species generation, NF-κB pathway activation, and osteoclastogenesis, ultimately impairing osteogenesis. AO polishing removed ~96.6% of powders from CP-Ti-AU-45, compared with 77.1% for Ti64, thereby minimizing potential particle- and ion-related inflammatory risks. Importantly, AO treatment generated a uniform and dense TiO_2_ layer with excellent biocompatibility. This oxide film not only suppressed uncontrolled Ti ion release but also provided a hydrophilic, negatively charged surface that facilitated hydroxyapatite nucleation and Ca-P mineral deposition [46,47]. Over time, this Ti-O-HA interfacial layer chemically bonded with newly formed bone, ensuring long-term osseointegration.

By removing inflammatory triggers (powders) and stabilizing the surface chemistry, AO polishing mitigated the pro-inflammatory M1 macrophage phenotype and promoted M2 polarization. Simultaneously, the tailored micro/nanostructures regulated protein adsorption dynamics to form a bioactive “protein corona”, which promoted integrin-mediated focal adhesion and activated downstream FAK/MAPK signaling. These processes facilitated osteoblast spreading, induced the expression of osteogenic genes (Runx2 and Alp), and promoted ECM maturation and mineralization [48]. Titanium nanoparticles can induce osteoblast death by oxidative stress, thereby inhibiting bone formation. The mechanism may involve wear particles disrupting intracellular homeostasis, activating ferroptosis pathways including Lpcat3, and triggering inflammatory cascades [49,50]. The expression of the Lpcat3 gene was significantly reduced after high-voltage AO polishing, suggesting that suppression of ferroptosis-related signaling may contribute to the improved cellular response.

It should be acknowledged that the in vivo experiments were conducted with a relatively small sample size (*n* = 3 per group). While this sample size is consistent with comparable studies in the field, it may limit the detection of subtle inter-group differences. Future studies with larger cohorts are warranted to further validate these findings.

Collectively, the physical effects of optimized topography provided anchoring and guidance for osteoblasts, while the chemical effects of powder elimination and TiO_2_ stabilization reduced local toxicity and enhanced mineralization. These were further reinforced by favorable immunological conditions and osteogenic signaling. Rather than acting independently, these factors function in a mutually reinforcing, stepwise amplification process: microstructures establish initial adhesion and mechanical interlocking, surface chemistry supports mineral nucleation, and biological cues optimize the local inflammatory microenvironment and osteogenic differentiation. This integrated mechanism underpins the superior bone regeneration and osseointegration performance of CP-Ti-AU-45 implants compared with conventional counterparts (Fig. 10H).

## Conclusion

This study demonstrates that high-voltage AO polishing enables highly efficient removal of residual powders (96.6%) from complex gyroid porous titanium scaffolds while simultaneously constructing multiscale, biomimetic micro-nano architectures (honeycomb, serrated, and Haversian-like channels) directly on the surface. The polished implants retained an elastic modulus (3 to 10 GPa) and yield strength (40 to 210 MPa) within the bone-matching range while exhibiting markedly enhanced plasticity and energy absorption capacity, thereby providing more stable mechanical support for bone regeneration. The biomimetic interface created by AO polishing significantly promoted cell adhesion, proliferation, and mineralization, with the CP-Ti-AU-45 specimens displaying the strongest osteoinductive potential during a 21-d co-culture. In vivo analyses further confirmed that AO-polished CP-Ti-AU-45 implants effectively mitigated chronic inflammation caused by residual powders and substantially improved new bone mineral density (0.19 g/cm^3^), bone volume fraction (33.8%), and bone–implant interfacial integration. The molecular mechanism may involve the activation of ECM–receptor interactions, adhesion plaques, and PI3K–Akt signaling pathways while inhibiting ferroptosis-related signaling pathways. Collectively, high-voltage AO polishing not only achieves efficient powder removal and biomimetic surface functionalization of porous titanium implants but also enables systematic optimization of their mechanical and biological performance, offering new experimental evidence and conceptual guidance for the design and clinical translation of next-generation, patient-specific orthopedic implants.

## Data Availability

Experimental data of this study will be provided upon reasonable request.
