# Supplementary material for: Efficient Powder Removal and Micro-Nano Structural Formation in 3D-Printed Porous Titanium Implants for Enhanced Osteogenesis
Source: Biomater Res. 2026 Nov 5;30:0434. doi: 10.34133/bmr.0434 (PMC13735203; doi:10.34133/bmr.0434)
Supplement: Supplementary file 1 [file bmr.0434.f1.docx]

Efficient powder removal and micro-nano structural formation in 3D-printed porous titanium implants for enhanced osteogenesis
Jincheng Tang^1,2,3†^, Sijing Li^4†^, Yonglun Liu^5^, Cheng Liu^4^, Guoxian Pei^4^, Lin Wang^4^*, Lin Wang^4^*, Ming Yan^3,5,6^*, Dewei Zhao^1^*
 ^1^Department of Orthopedics, Affiliated Zhongshan Hospital of Dalian University, Dalian, 116001, China
^2^School of Materials Science and Engineering, Harbin Institute of Technology, Harbin, 150001, China
^3^Department of Materials Science and Engineering, Southern University of Science and Technology, Shenzhen, 518055, China
^4^School of Medicine, Southern University of Science and Technology, Shenzhen, 518055, China
^5^3R Additive Technology (Shenzhen) Co.,Ltd. Shenzhen, 518055, China
^6^Jiaxing Research Institute, Southern University of Science and Technology, Jiaxing 314031, China
†These two authors contributed to the study equally.
*Corresponding authors, email address: [zhaodewei2000@163.com;](mailto:zhaodewei2000@163.com;) yanm@sustech.edu.cn; wangl7@sustech.edu.cn

**Fig. S1.** Schematic diagram of (A) sample printing direction and (B) scanning strategy.

**Table S1** Printing parameters of gyroid porous CP-Ti and Ti64 samples.

| Sample | Laser power (W) | Scanning speed (mm/s) | Hatch spacing (μm) | Layer thickness (μm) | Volume energy density (J/mm^3^) |
| --- | --- | --- | --- | --- | --- |
| CP-Ti-35 | 158 | 1500 | 100 | 30 | 35 |
| CP-Ti-40 | 180 | 1500 | 100 | 30 | 40 |
| CP-Ti-45 | 203 | 1500 | 100 | 30 | 45 |
| CP-Ti-50 | 225 | 1500 | 100 | 30 | 50 |
| Ti64-35 | 158 | 1500 | 100 | 30 | 35 |
| Ti64-40 | 180 | 1500 | 100 | 30 | 40 |
| Ti64-45 | 203 | 1500 | 100 | 30 | 45 |
| Ti64-50 | 225 | 1500 | 100 | 30 | 50 |

**Fig. S2.** (A) Schematic diagram of high-voltage anodic oxidation polishing; (B) Corresponding parameters employed in this study

**Table S2** Relative densities and average grain sizes of gyroid porous CP-Ti and Ti64 samples under different printing parameters.

| Sample | Volume energy density (J/mm^3^) | Relative density (%) | Average grain size (μm) |
| --- | --- | --- | --- |
| CP-Ti-35 | 35 | 98.96 | 2.60 |
| CP-Ti-40 | 40 | 99.54 | 2.83 |
| CP-Ti-45 | 45 | 99.82 | 3.06 |
| CP-Ti-50 | 50 | 99.85 | 3.26 |
| Ti64-35 | 35 | 99.33 | 2.25 |
| Ti64-40 | 40 | 99.46 | 2.34 |
| Ti64-45 | 45 | 99.74 | 2.47 |
| Ti64-50 | 50 | 99.77 | 2.52 |

**Fig. S3.** 3D and 2D planar views of the surface roughness of gyroid porous samples.

**Fig. S4**. Detailed XPS spectra of Al and V on the surface of gyroid porous (A) Ti64-45, (B) Ti64-A-45, and (C) Ti64-AU-45 sample.

**Table S3** Compressive properties of gyroid porous CP-Ti samples under different printing parameters.

| Samples | Yield strength (MPa) | Ultimate compressive strength (MPa) | Plateau Stress (MPa) | Energy Absorption (MJ/m^3^) | Elasticity modulus (GPa) | Elongation (%) |
| --- | --- | --- | --- | --- | --- | --- |
| CP-Ti-35 | 80.79 | 210.22 | 108.09 | 59.50 | 6.57 | 64.92 |
| CP-Ti-40 | 93.24 | 257.20 | 128.75 | 72.23 | 7.81 | 65.54 |
| CP-Ti-45 | 104.13 | ＞2000 | 188.87 | 101.74 | 8.46 | ＞80 |
| CP-Ti-50 | 113.72 | ＞2000 | 207.41 | 113.01 | 8.82 | ＞80 |
| CP-Ti-AU-35 | 67.77 | ＞2000 | 111.06 | 57.75 | 5.97 | ＞80 |
| CP-Ti-AU-40 | 82.06 | ＞2000 | 131.44 | 69.73 | 6.71 | ＞80 |
| CP-Ti-AU-45 | 97.62 | ＞2000 | 183.64 | 90.72 | 7.81 | ＞80 |
| CP-Ti-AU-50 | 103.68 | ＞2000 | 204.73 | 98.95 | 8.22 | ＞80 |

**Table S4** Compressive properties of gyroid porous Ti64 samples under different printing parameters.

| Samples | Yield strength (MPa) | Ultimate compressive strength (MPa) | Plateau Stress (MPa) | Energy Absorption (MJ/m^3^) | Elasticity modulus (GPa) | Elongation (%) |
| --- | --- | --- | --- | --- | --- | --- |
| Ti64-35 | 155.43 | 221.20 | - | 64.70 | 7.81 | 38.08 |
| Ti64-40 | 180.73 | 235.78 | - | 80.96 | 8.05 | 45.97 |
| Ti64-45 | 216.13 | 272.33 | - | 106.60 | 9.82 | 48.67 |
| Ti64-50 | 234.29 | 285.60 | - | 102.43 | 10.09 | 43.84 |
| Ti64-AU-35 | 128.69 | 188.71 | - | 81.13 | 7.16 | 49.68 |
| Ti64-AU-40 | 138.96 | 200.08 | - | 81.49 | 7.48 | 49.61 |
| Ti64-AU-45 | 192.44 | 258.49 | - | 137.18 | 8.54 | 61.05 |
| Ti64-AU-50 | 208.74 | 281.15 | - | 100.48 | 9.22 | 43.47 |

Tensile specimens fabricated under different volumetric energy density (VED) conditions were cut using wire electrical discharge machining, followed by surface grinding prior to tensile testing. The stress-strain curves of CP-Ti and Ti64 specimens (Fig. S9 and Table S5) demonstrate that porous tensile samples prepared at different VEDs exhibit pronounced differences in both ultimate tensile strength and elongation.

With increasing VED, both the stress level and the corresponding deformation capability (ductility) of the specimens show a clear upward trend (Fig. S9A). A higher VED significantly enhances the densification degree and metallurgical bonding quality of the samples, thereby improving their tensile mechanical performance. When the VED was set to 45 J/mm^3^, the CP-Ti-45 specimen achieved an elongation of 10.75%, indicating favorable toughness and ductility. As the VED further increased to 50 J/mm^3^, the CP-Ti-50 specimen reached a maximum ultimate tensile strength of 152.15 MPa. Although the elongation did not reach its maximum at this condition, the overall mechanical performance remained superior, reflecting a reasonable balance between strength and plasticity.

In comparison, Ti64 specimens exhibited a stress–strain evolution trend similar to that of CP-Ti under varying VED conditions, whereby improvements in densification and metallurgical bonding with increasing VED led to enhanced mechanical properties (Fig. S9C). However, owing to the strengthening effect of alloying elements, Ti64 generally demonstrated higher tensile strength but relatively lower ductility. The optimal elongation and ultimate tensile strength of Ti64 were also obtained at VEDs of 45 J/mm^3^ and 50 J/mm^3^, respectively. Fracture surface analysis revealed that Ti64 exhibited relatively sparse dimple structures, indicating limited plastic deformation capability (Fig. S9D), and suggesting that its mechanical behavior is predominantly characterized by high strength and low ductility.

SEM fracture surface observations further elucidate the fracture mechanisms of specimens fabricated under different VED conditions (Fig. S10). At lower VEDs (CP-Ti-35 and CP-Ti-40), the fracture surfaces are characterized by pronounced pores, loosely bonded regions, and rough irregular cleavage-like features. These defects result in severe stress concentration and insufficient plastic deformation, ultimately leading to inferior strength and ductility (Figs. S10A-D). With increasing VED (CP-Ti-45 and CP-Ti-50), the fracture surfaces gradually transform into a ductile fracture mode dominated by abundant fine dimples, with more uniformly distributed microvoids and a significant reduction in large pores and particle-related defects (Figs. S10E-H). This dimple-dominated fracture behavior indicates improved metallurgical bonding and enhanced intergranular cohesion, thereby contributing to the simultaneous improvement of strength and ductility.

Nevertheless, notable differences in fracture morphology are still observed between CP-Ti and Ti64. SEM analysis shows that CP-Ti exhibits deeper and more pronounced dimples, suggesting a more extensive and uniform microvoid nucleation, growth, and coalescence process during plastic deformation, which is indicative of superior ductility. In contrast, Ti64 presents relatively sparser dimples with variations in depth and size (Fig. S11). As the VED increases, the dimples on the fracture surfaces of Ti64-45 and Ti64-50 specimens become progressively denser and deeper (Figs. S11E-H), indicating that their fracture behavior is also strongly influenced by improvements in metallurgical bonding and microstructural evolution.

Overall, 3D-printed CP-Ti fabricated under different VED conditions exhibits significant variations in microstructure, densification level, and grain bonding characteristics, which collectively govern the stress–strain response during tensile loading. The results of this study demonstrate that a VED of 45 J/mm^3^ enables CP-Ti and Ti64 to achieve an optimal balance between strength and ductility, rendering them not only mechanically competent but also more suitable for subsequent in vitro and in vivo biological evaluations as well as orthopedic porous implant applications.

**Fig. S5**. (A, C) Tensile stress-strain curves and (B, D) post-tensile test sample images of gyroid porous CP-Ti and Ti64 samples under different printing parameters.

**Fig. S6**. SEM cross-sectional morphology of gyroid porous CP-Ti-35, CP-Ti-40, CP-Ti-45, and CP-Ti-50 samples under different printing.

**Fig. S7**. SEM cross-sectional morphology of gyroid porous Ti64-35, Ti64-40, Ti64-45, and Ti64-50 samples under different printing.

**Table S5** Tensile properties of gyroid porous CP-Ti and Ti64 samples under different printing parameters.

| Samples | Yield strength (MPa) | Ultimate tensile strength (MPa) | Elasticity modulus (GPa) | Elongation (%) |
| --- | --- | --- | --- | --- |
| CP-Ti-35 | 73.40 | 108.60 | 17.98 | 8.17 |
| CP-Ti-40 | 89.43 | 122.10 | 18.39 | 8.54 |
| CP-Ti-45 | 102.29 | 142.77 | 19.74 | 10.75 |
| CP-Ti-50 | 107.37 | 152.15 | 20.32 | 8.01 |
| Ti64-35 | 156.20 | 220.09 | 21.23 | 3.17 |
| Ti64-40 | 188.52 | 256.44 | 23.67 | 3.70 |
| Ti64-45 | 200.43 | 280.01 | 27.20 | 3.81 |
| Ti64-50 | 229.54 | 310.83 | 29.61 | 3.52 |

**Fig. S8**. Schematic diagram of the PI3K-Akt signaling pathway in MC3T3-E1 cells after incubation for 3 days with polished gyroid porous CP-Ti-AU-45 samples.

**Fig. S9**. The molecular mechanism of high-voltage anodic oxidation. (A) Volcano plot displaying the differentially expressed genes between the Ti64-AU-45 and Ti64-45 groups. (B) Protein-protein interaction (PPI) network of representative DEGs, highlighting the core clusters related to osteogenesis and cell adhesion. (C) KEGG pathway enrichment analysis showing the top signaling pathways influenced by the surface modification. (D) Quantitative comparison of mRNA expression levels for key osteogenic markers. (E) GO enrichment analysis categorized by MF, CC, and BP. Data are presented as mean ± SD (**p* < 0.05, ***p* < 0.01, ****p* < 0.001).

**Fig. S10**. Enlarged images of selected regions showing residual powders in (A) Methylene Blue-stained and (B) Toluidine Blue-stained sections.

**Table S6** Comparison of relative density, mechanical properties, and porosity of gyroid porous CP-Ti-45, CP-Ti-AU-45, Ti64-45, and Ti64-AU-45 samples before and after polishing.

| Samples | Relative density (*ρ*^*^/*ρ_s_*) | Relative elastic modulus (*E*^*^/*E_s_*) | Relative yield strength (*σ*^*^/*σ_s_*) | Actual porosity (%) |
| --- | --- | --- | --- | --- |
| CP-Ti-45 | 0.468 | 0.077 | 0.195 | 53.2 |
| CP-Ti-AU-45 | 0.403 | 0.071 | 0.183 | 59.7 |
| Ti64-45 | 0.471 | 0.089 | 0.213 | 52.9 |
| Ti64-AU-45 | 0.413 | 0.078 | 0.189 | 58.7 |
